# Functional Differentiation of Cyclins and Cyclin-Dependent Kinases in Giardia lamblia

**DOI:** 10.1128/spectrum.04919-22

**Published:** 2023-03-06

**Authors:** Juri Kim, Eun-Ah Park, Mee Young Shin, Soon-Jung Park

**Affiliations:** a Department of Tropical Medicine, Yonsei University College of Medicine, Seoul, South Korea; Weill Cornell Medicine

**Keywords:** *Giardia lamblia*, cyclin-dependent kinase, cyclin, cell cycle, flagella

## Abstract

Cyclin-dependent kinases (CDKs) are serine/threonine kinases that control the eukaryotic cell cycle. Limited information is available on Giardia lamblia CDKs (GlCDKs), GlCDK1 and GlCDK2. After treatment with the CDK inhibitor flavopiridol-HCl (FH), division of Giardia trophozoites was transiently arrested at the G1/S phase and finally at the G2/M phase. The percentage of cells arrested during prophase or cytokinesis increased, whereas DNA synthesis was not affected by FH treatment. Morpholino-mediated depletion of GlCDK1 caused arrest at the G2/M phase, while GlCDK2 depletion resulted in an increase in the number of cells arrested at the G1/S phase and cells defective in mitosis and cytokinesis. Coimmunoprecipitation experiments with GlCDKs and the nine putative G. lamblia cyclins (Glcyclins) identified Glcyclins 3977/14488/17505 and 22394/6584 as cognate partners of GlCDK1 and GlCDK2, respectively. Morpholino-based knockdown of Glcyclin 3977 or 22394/6584 arrested cells in the G2/M phase or G1/S phase, respectively. Interestingly, GlCDK1- and Glcyclin 3977-depleted Giardia showed significant flagellar extension. Altogether, our results suggest that GlCDK1/Glcyclin 3977 plays an important role in the later stages of cell cycle control and in flagellar biogenesis. In contrast, GlCDK2 along with Glcyclin 22394 and 6584 functions from the early stages of the Giardia cell cycle.

**IMPORTANCE** Giardia lamblia CDKs (GlCDKs) and their cognate cyclins have not yet been studied. In this study, the functional roles of GlCDK1 and GlCDK2 were distinguished using morpholino-mediated knockdown and coimmunoprecipitation. GlCDK1 with Glcyclin 3977 plays a role in flagellum formation as well as cell cycle control of G. lamblia, whereas GlCDK2 with Glcyclin 22394/6584 is involved in cell cycle control.

## INTRODUCTION

Giardia lamblia is a pathogen that causes diarrheal disease and exists as either cysts or trophozoites. Trophozoites, the multiplying form of G. lamblia found in hosts, have two nuclei and distinct cytoskeletal structures: a ventral disc, median body, and four pairs of flagella (1). The mechanisms involved in the regulation Giardia trophozoite division remain unknown. Live imaging indicated that cytokinesis occurs 60 times faster in Giardia cells than in mammalian cells, making it difficult to observe dividing cells (2). During cytokinesis, G. lamblia trophozoites use flagellum-mediated membrane tension rather than the typical myosin-dependent contractile rings that are used for daughter cell separation in mammalian cells. Giardia cells are predominantly seen in the G2/M phase in *in vitro* cultures (3). The expression of phase-specific Giardia proteins has been shown using synchronized cell cultures with chemical treatment or counterflow centrifugal elutriation (3–6). The cell cycle of Giardia can proceed despite blocking DNA synthesis, defective mitotic spindles, or double-stranded DNA breaks; therefore, it may have an incomplete checkpoint system to regulate its cell cycle (7). In addition, Giardia lacks the anaphase-promoting complex (APC) required for ubiquitin-dependent degradation of cell cycle components (7, 8).

In mammals, cell division is a well-organized and complex process involving macromolecular machinery and numerous protein interactions. This process is dynamic and finely controlled by interconnected signaling pathways, including the cyclin-dependent kinase (CDK), aurora kinase (AK), and polo-like kinase (PLK) (9, 10). In Giardia, the role of AK in cell division has been proven using AK-specific inhibitors (11), the function of which is related to phosphorylation of G. lamblia end-binding protein 1, a microtubule-binding protein involved in cell cycle control in eukaryotes (12). Recently, a putative PLK in G. lamblia was demonstrated to play a role in cytokinesis and flagellar biogenesis using PLK inhibitors and morpholino-based knockdown experiments (13).

In mammals, CDK is a key regulator of the cell cycle and has 20 paralogues (14). The activation of these Ser/Thr kinases is modulated, in part, by binding to their cognate cyclins (15). In addition, activation of the CDK-cyclin complex is finely controlled via a series of phosphorylations of critical residues that are controlled by CDK kinase and CDK inhibitors (16). The budding yeast, Saccharomyces cerevisiae, has six CDKs, but only Cdc28 is required for mitotic and meiotic cell cycle control (17). Unlike mitotic kinases such as AKs and PLKs, CDKs play a role in DNA synthesis (18). Among the several putative open reading frames (ORFs) for CDKs in the GiardiaDB resource, only two ORFs (GL50803_8037 and GL50803_16802) have the PSTAIRE motif for cyclin binding and were named G. lamblia CDK1 (GlCDK1) and GlCDK2, respectively (19). GlCDK2 has been shown to have kinase activity toward the transcription factor Myb2, whose expression is induced during encystation (20, 21). In addition, GlCDK1 was found to interact with G. lamblia cyclin (Glcyclin) 3977 (GL50803_3977), and immunoprecipitates of GlCDK1-Glcyclin 3977 could phosphorylate bovine histone H1 (8).

In this study, the roles of GlCDK1 and GlCDK2 in Giardia cell division and flagellar biogenesis were examined using a CDK inhibitor and morpholino-mediated knockdown. The binding activity of these GlCDKs to nine putative Glcyclins was examined via coimmunoprecipitation (co-IP), and the co-IP extracts were tested for kinase activity to bovine histone H1, providing information on the cognate Glcyclins for each GlCDK.

## RESULTS

### Effect of CDK inhibition on the cell cycle of Giardia.

Two different pan-CDK inhibitors (flavopiridol-HCl [FH] and Dinaciclib [22]) and one CDK2-specific inhibitor, K03861 (23) were used to define the role of CDKs in Giardia cell division (data not shown). Flow cytometric analyses of the DNA content of Giardia cells indicated that FH induced more distinct arrest of Giardia cells in the G2/M phase than Dinaciclib. In order to obtain an arrest of Giardia cells in the G2/M phase, a shorter time, such as 3 h, was required with 2.5 μM FH than K03861 (24 h). Therefore, FH was used for further studies.

Giardia trophozoites were treated for 24 h with various concentrations of FH (1.0 to 10 μM), and the DNA content of FH-treated cells was analyzed by flow cytometry (see Fig. S1A in the supplemental material). The proportion of G2/M-phase cells increased with FH treatment, and the DNA peak shifted to the right in cells treated with a higher FH concentration (>2.5 μM). In the control cells treated with 0.1% dimethyl sulfoxide (DMSO), a mixture of G1/S-phase (19%) and G2/M-phase cells (81%) was observed. The number of G2/M-phase cells increased to 91%, whereas the number of G1/S-phase cells decreased to 9% when treated with 2.5 μM FH. G. lamblia trophozoites were then treated with 0.025% DMSO or 2.5 μM FH for various durations (1 to 24 h) and then analyzed by flow cytometry (Fig. S1B). Cells treated with 2.5 μM FH for 3 h demonstrated the most dramatic arrest at the G2/M phase and were used for further studies.

**FIG 1:**
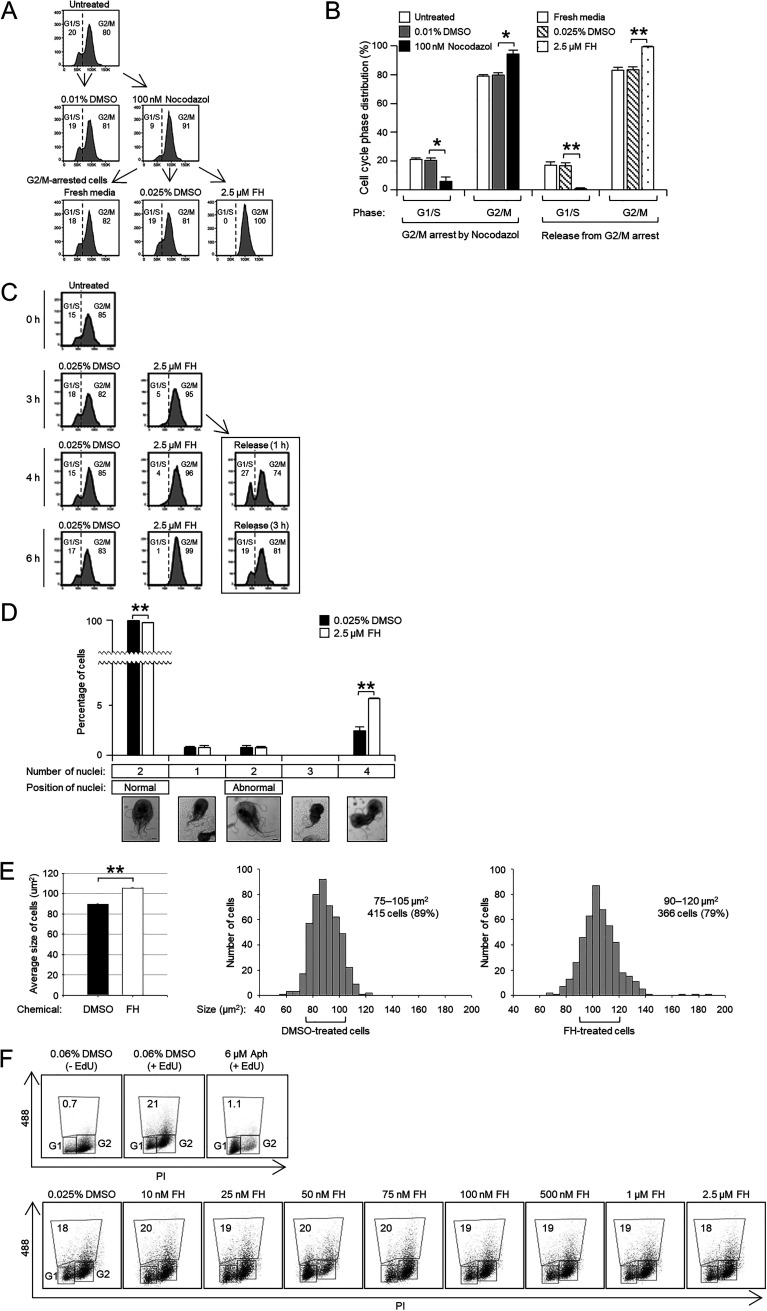
Effect of the cyclin-dependent kinase (CDK) inhibitor, flavopiridol-HCl (FH) on cell division in Giardia. (A and B) Giardia trophozoites pretreated with 100 nM nocodazole for 2 h were incubated in the fresh medium alone, with 0.025% dimethyl sulfoxide (DMSO) or with 2.5 μM FH for 3 h. Untreated cells and cells exposed to 0.01% DMSO were prepared as controls for the arrested cells by nocodazole. The DNA content of these cells was measured using flow cytometry. (A) Representative histograms for each phase of cell cycle. (B) Bar graph showing the average values of 3 independent cultures. (C) Giardia trophozoites were treated with 2.5 μM FH for 3 h and then transferred into the medium without FH. As controls, Giardia cells were treated with 0.025% DMSO or 2.5 μM FH. The DNA content of these cells was measured using flow cytometry. Representative histograms are presented among three independent experiments. (D) Giardia trophozoites were treated with 2.5 μM FH (open bars) or 0.025% DMSO (closed bars) for 3 h and stained with Giemsa. At least 1,000 cells were examined to evaluate the number and position of the nuclei for each condition. A representative cell for each category is shown. (E) The size of cells treated with 2.5 μM FH (open bars) or 0.025% DMSO (closed bars) was determined using Zen imaging software. The sizes of these two populations are shown as a histogram. The data shown are representative of three independent replicates. (F) Effect of the CDK inhibitor, FH, on DNA synthesis in G. lamblia. Giardia cells were incubated either with DMSO (0.06%) or aphidicolin (6 μM) for 6 h under dark conditions as a control. Giardia trophozoites were incubated with various concentrations of FH (10 nM to 2.5 μM) for 1.5 h. Control cells were treated with 0.025% DMSO. These cells were then treated with 50 μM 5-ethynyl-2′-deoxyuridine (EdU), 1.5 μM iFluor 488 azide, and propidium iodide (PI). The percentages of cells stained with EdU and PI were determined by flow cytometry. Data shown are representative of three independent replicates. *, *P* < 0.05; **, *P* < 0.01.

Giardia cells treated with nocodazole were predominantly arrested at the G2/M phase (94%). When these cells were incubated in fresh medium without any supplement or with 0.025% DMSO, a portion of cells (17%) shifted into the G1/S-phase (Fig. 1A and B). In contrast, most of the cells (99%) remained in the G2/M phase when the cells were cultured in medium containing 2.5 μM FH.

When the FH-arrested cells at the G2/M phase were incubated in fresh medium without FH, the percentages of Giardia cells at the G1/S and G2/M phases were 19% and 81% at 3 h postrelease, respectively, which are the same with the control cells (Fig. 1C). This result indicated that FH blocked the Giardia cell cycle in the G2/M phase, and the arrest was reversible with the removal of FH.

Cells treated with 2.5 μM FH for 3 h were also stained with Giemsa. More than 1,000 stained cells were counted for the number and position of nuclei as follows: one nucleus, two nuclei in the normal position, two nuclei in the abnormal position, three nuclei, or four nuclei (Fig. 1D). Most control cells had two nuclei in the normal position (97%), and the percentage of these cells decreased slightly to 92% when the cells were treated with 2.5 μM FH for 3 h. The numbers of cells with one nucleus or two abnormally positioned nuclei were similar in the FH-treated cells compared to those in the control group. The number of cells undergoing cytokinesis significantly increased to 5.9% in FH-treated cells from 1.3% in the control cells. Interestingly, Giardia trophozoites with condensed nuclei, which were larger and more deeply stained, were found more frequently among the FH-treated cells (0.8%) than the untreated cells (0.5%).

Next, we examined whether CDK inhibition affected the size of Giardia by flow cytometric analysis of cells whose membranes were fluorescently labeled (Fig. 1E). Control cells treated with 0.025% DMSO had sizes between 75 and 105 μm^2^ with an average of 90 μm^2^. When the cells were treated with 2.5 μM FH, 75% of the counted cells were 90 to 120 μm^2^ in area with an average of 105 μm^2^. Therefore, FH-mediated inhibition of CDKs resulted in enlargement of Giardia cells compared with the untreated cells.

In mammalian cells, CDKs play an important role in DNA replication (24). To determine the concentration of FH needed to cause G1/S-phase arrest, Giardia trophozoites were treated for 1 h with various concentrations of FH (10 nM to 1 μM), and the DNA content of the FH-treated cells was analyzed using flow cytometry (Fig. S1C). The proportion of G1/S-phase cells increased from 24% to 36% in cells treated with 50 nM FH. Next, G. lamblia trophozoites were treated with 0.0005% DMSO or 50 nM FH for various durations (0.5 to 3 h) and analyzed by flow cytometry (Fig. S1D). The increase in G1/S-phase cells was transient, and cells treated with 50 nM FH for 1.5 h showed a maximal increase (83%) from that of DMSO-treated cells (22 to 26%). Therefore, this incubation time (1.5 h) was used in subsequent studies.

The role of Giardia CDKs in DNA synthesis was examined by comparing 5-ethynyl-2′-deoxyuridine (EdU) incorporation between control cells (0.025% DMSO) and FH-treated cells (10 nM to 2.5 μM) for 1.5 h (Fig. 1F). Giardia cells treated with 6 μM aphidicolin, an inhibitor of DNA replication, were used as a positive control for G1/S-arrested cells, in which EdU incorporation dramatically decreased to 1.1% from 21% in DMSO-treated cells. However, FH-treated cells at any concentration did not show alterations in EdU incorporation (18 to 20%) compared to control DMSO-treated cells (19%).

### Effect of GlCDK knockdown on cell division in Giardia.

A homology search in GiardiaDB (https://giardiadb.org/giardiadb/app, release 60, accessed 9 November 2022) indicated two ORFs (GL50803_8037 and GL50803_16802) as putative G. lamblia CDKs, which have a PSTAIRE cyclin-binding domain and form valid protein structures as determined using AlphaFold. They are conserved in the genomes of various assemblages of G. lamblia (assemblages A, B, and E [25–27], respectively) as well as in Giardia muris (28). These ORFs were predicted to encode a 35-kDa protein with an pI of 6.5 for GlCDK1 and a 33-kDa protein with a pI of 9.0 for GlCDK2. A search of domains within these ORFs using the Entrez program (http://www.expasy.org/) indicated that they contain a serine-threonine kinase domain from amino acid residues 14 to 301 for GlCDK1 and from amino acids 7 to 289 for GlCDK2.

**FIG 2:**
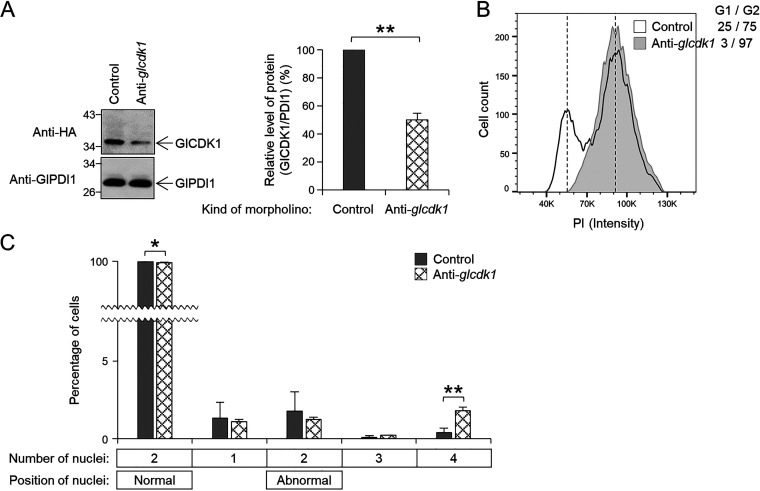
Effect of morpholino-mediated GlCDK1 knockdown on cell division in Giardia cells. Giardia cells expressing HA-tagged GlCDK1 were collected at 12 h after transfection with control (gray bars) or anti-*glcdk1* (checked bars) morpholinos. (A) Morpholino-mediated GlCDK1 knockdown in Giardia. Bar graph of relative expression of HA-tagged GlCDK1 in cells treated with anti-*glcdk1* morpholinos compared with that in the control cells. (B and C) Effect of morpholino-mediated GlCDK1 knockdown on (B) DNA content and (C) nuclear phenotypes of G. lamblia. Data are presented as the mean of three independent experiments. *, *P* < 0.05; **, *P < *0.01.

The plasmids pGlCDK1HA.NEO and pGlCDK2HA.NEO were constructed and used to generate transgenic Giardia cells expressing hemagglutinin (HA)-tagged GlCDKs (Table S1). Western blotting of the resulting Giardia cell extracts confirmed the expression of HA-tagged GlCDK1 and GlCDK2 as immunoreactive bands (data not shown), and these cells were used in subsequent experiments. To determine the role of GlCDK1 in Giardia, we designed an anti-*glcdk1* morpholino to block the expression of GlCDK1 protein (Table S2). When the cells were collected at 6 to 24 h posttransfection with the anti-*glcdk1* morpholino and analyzed for inhibition of GlCDK1 expression by Western blotting, the cells at 12 h posttransfection demonstrated maximum inhibition of GlCDK1 expression (50%; Fig. 2A). In GlCDK1-depleted cells, the number of cells in the G2/M phase increased to 97% compared to 75% in control cells transfected with a control morpholino (Fig. 2B). The effect of GlCDK1 knockdown on cell division was investigated based on the number of nuclei and the percentage of dividing cells. The proportion of cells with two normally positioned nuclei decreased slightly from 98% to 96%. The percentages of cells with one nucleus, two nuclei in an abnormal position, or three nuclei were not altered in cells treated with an anti-*glcdk1* morpholino (Fig. 2C). The proportion of cells undergoing cytokinesis increased in GlCDK1-depleted cells.

**FIG 3:**
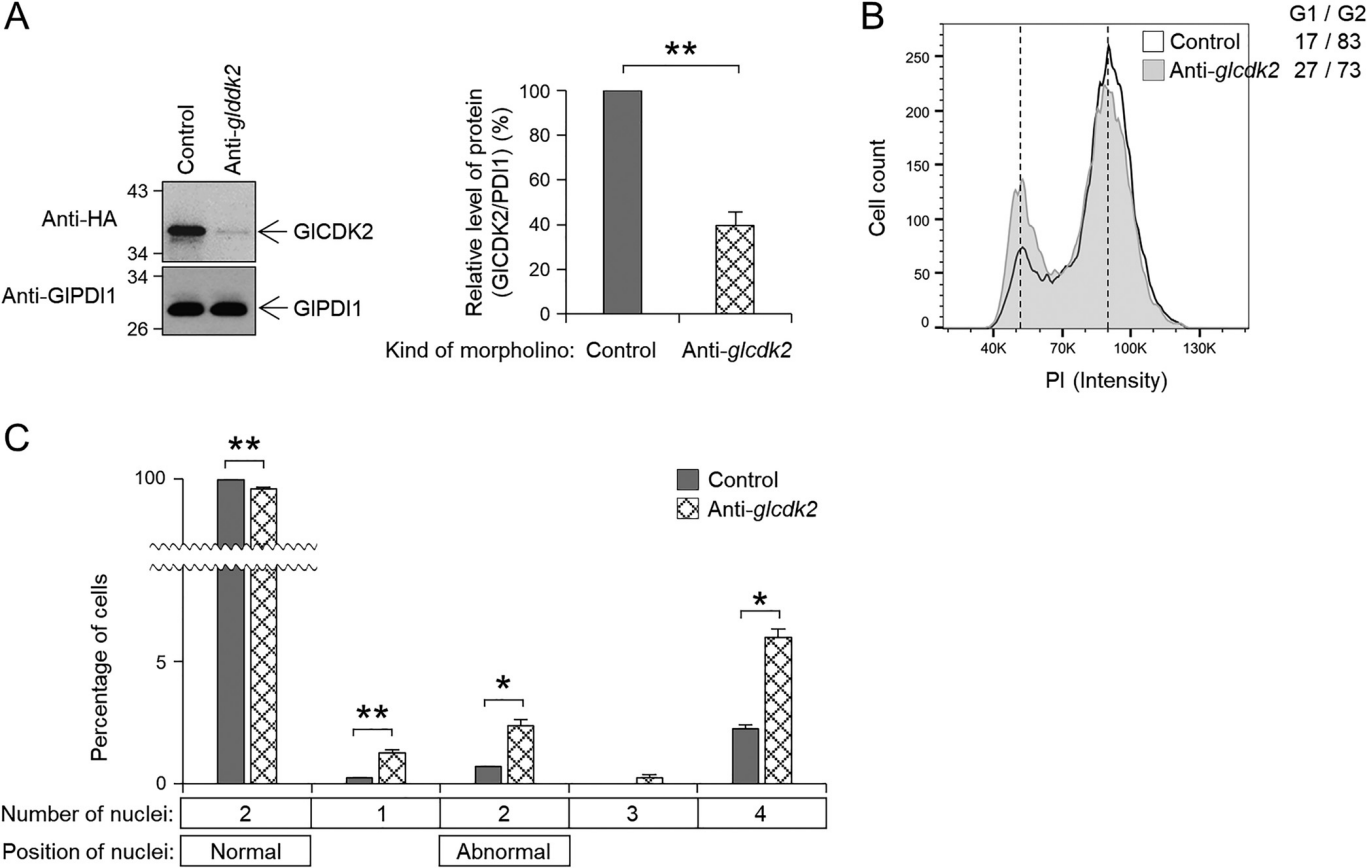
Effect of morpholino-mediated GlCDK2 knockdown on cell division in Giardia. Giardia cells expressing HA-tagged GlCDK2 were collected at 6 h after transfection with control (gray bars) or anti-*glcdk2* (checked bars) morpholinos. (A) Morpholino-mediated GlCDK2 knockdown in Giardia. Bar graph of relative expression of HA-tagged GlCDK2 in cells treated with anti-*glcdk2* morpholino compared with that in the control cells. (B and C) Effect of morpholino-mediated GlCDK2 knockdown on (B) DNA content and (C) nuclear phenotypes of G. lamblia. Data are presented as the mean of three independent experiments. *, *P* < 0.05; **, *P < *0.01.

GlCDK2 depletion was detected by Western blotting in Giardia cells at 6 to 24 h posttransfection with an anti-*glcdk2* morpholino. The expression of HA-tagged GlCDK2 decreased to 40% at 6 h posttransfection (Fig. 3A), resulting in an increase in the population at the G1/S phase from 17% to 27% (Fig. 3B). After transfection (6 h) with the anti-*glcdk2* morpholino, the percentage of cells with two normally positioned nuclei decreased from 98% to 85%. The percentage of cells with one or two nuclei in an abnormal position increased in cells transfected with anti-*glcdk2* morpholino (Fig. 3C). Analysis of cells undergoing cytokinesis showed a statistically significant increase to 8.1% from 1.3% of the control cells. Interestingly, the number of cells with condensed DNA increased to 5.0% among the GlCDK2-depleted cells from 0.5% of the control cells.

We then examined whether GlCDK depletion affects DNA synthesis in Giardia cells by measuring EdU incorporation. GlCDK1-depleted cells clearly demonstrated cell cycle arrest at the G2/M phase but did not show a dramatic change in EdU incorporation (Fig. S2A). Cells at 6 h posttransfection with anti-*glcdk2* morpholino demonstrated an increased population in the G1/S phase, whereas no change in EdU incorporation (20%) was detected between GlCDK2-depleted and control cells (Fig. S2B).

### Localization of GlCDKs in interphase and dividing Giardia cells.

**FIG 4:**
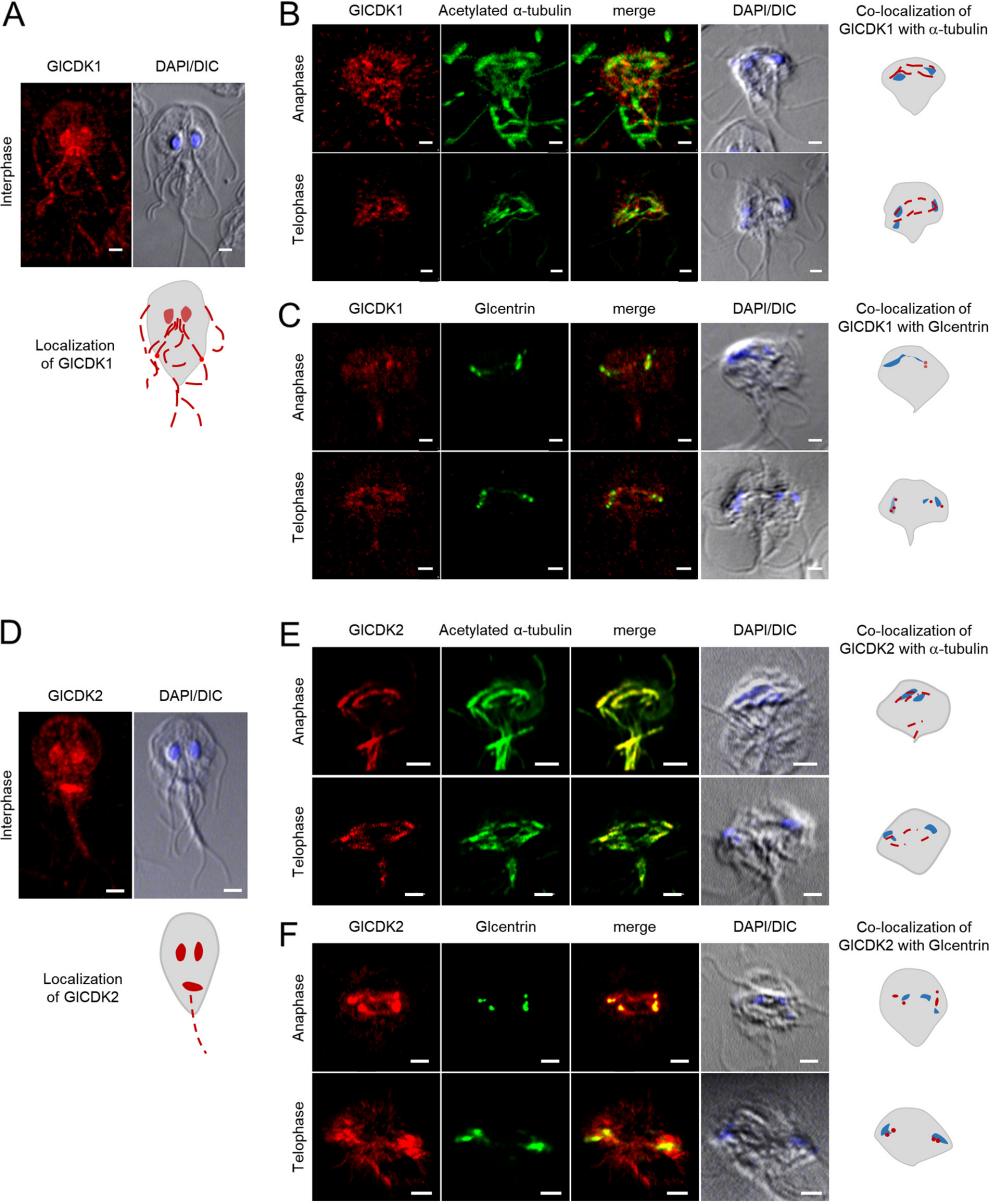
Expression and localization of GlCDK1 and GlCDK2 in Giardia expressing HA-tagged GlCDKs. (A to C) Localization of GlCDK1 in Giardia. (A) IFA of interphase Giardia using anti-HA antibodies. (B) IFA of Giardia in anaphase or telophase stained with anti-HA and anti-acetylated-α-tubulin antibodies. (C) IFA of Giardia in anaphase or telophase stained with anti-HA and anti-Glcentrin antibodies. (D to F) Localization of GlCDK2 in Giardia. (D) IFA of interphase Giardia using anti-HA antibodies. (E) IFA of Giardia in anaphase or telophase stained with anti-HA and anti-acetylated-α-tubulin antibodies. (F) IFA of Giardia in anaphase or telophase stained with anti-HA and anti-Glcentrin antibodies. The cell morphology is represented by differential interference contrast (DIC) images. Scale bars = 2 μm.

Transgenic Giardia cells expressing HA-tagged GlCDK1 were used for immunofluorescence assays (IFAs) (Fig. 4A). Interphase Giardia trophozoites labeled with anti-HA antibodies revealed HA-tagged GlCDK1 expression in the nucleus, axonemes, flagella, and two pores for the posterolateral flagella (Fig. 4A). The Giardia cells were also costained with anti-HA and anti-acetylated-α-tubulin antibodies (Fig. 4B). Giardia cells in anaphase (*n* = 10) and telophase (*n* = 7) showed that GlCDK1 was present in the mitotic spindles and axonemes. Double staining of dividing Giardia cells in anaphase (*n* = 13) and telophase (*n* = 11) with anti-HA and anti-Glcentrin (GL50803_104685) antibodies indicated the localization of GlCDK1 at basal bodies in the dividing cells (Fig. 4C).

Staining of interphase Giardia cells carrying pGlCDK2HA.NEO with anti-HA antibodies (Fig. 4D) showed localization of GlCDK2 in the nucleus, median body, and caudal flagella (*n* = 9). Costaining of these cells at the anaphase and telophase (*n* = 10 and 14, respectively) with anti-HA/anti-acetylated-α-tubulin antibodies (Fig. 4E) or anti-HA/anti-Glcentrin antibodies (Fig. 4F) revealed distinct localization of GlCDK2 at mitotic spindles and basal bodies, respectively.

### Effect of GlCDK overexpression on Giardia growth.

**FIG 5:**
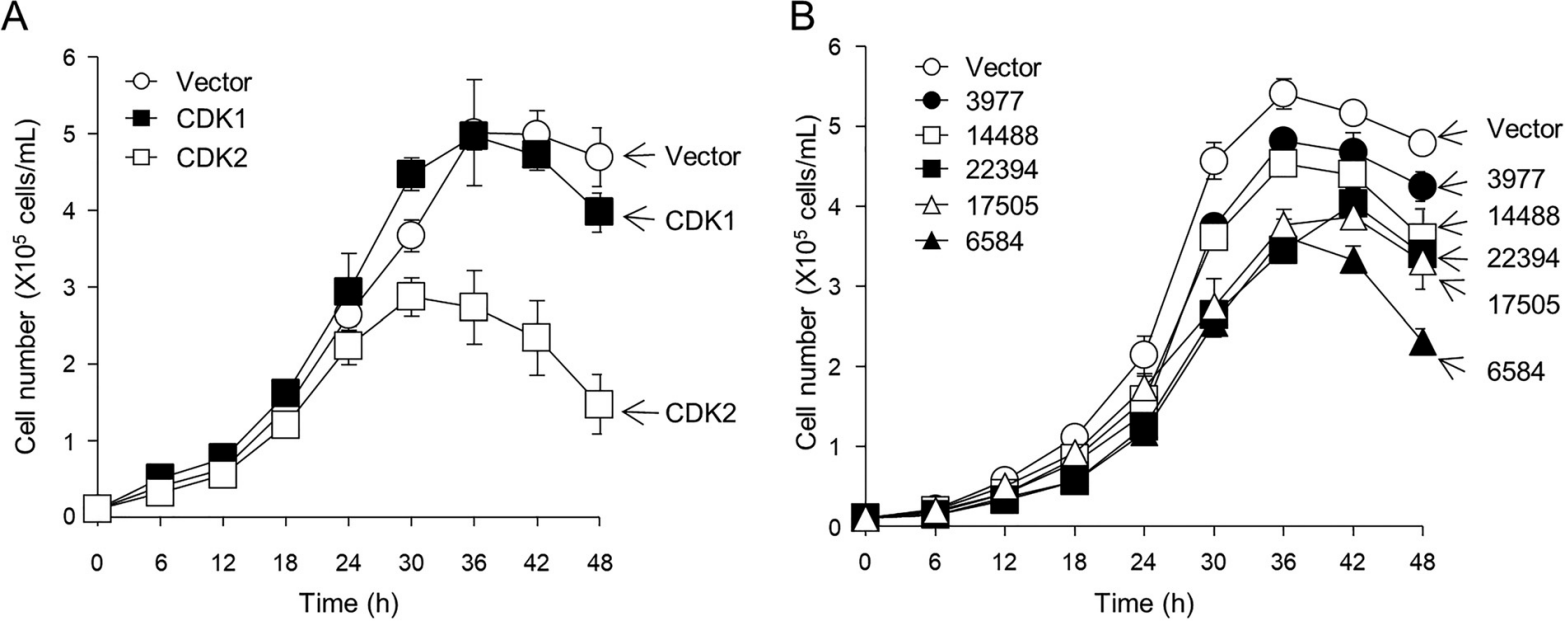
Effects of overexpression of GlCDKs and G. lamblia cyclins (Glcyclins) on Giardia growth. (A) Growth curves of Giardia carrying the vector plasmid (open circles) and expressing GlCDK1 (closed squares) or GlCDK2 (open squares). (B) Growth curves of Giardia carrying the vector plasmid (open circles) or expressing Glcyclins 3977 (closed circles), 14488 (open squares), 22394 (closed squares), 17505 (open triangles), or 6584 (closed triangles). Each experiment comprised three cultures and was repeated three times using independent transfectants. The number of cells per milliliter was counted using a hemocytometer. Data are presented as means ± SD of three independent experiments.

Giardia cells carrying pGlCDK1HA.NEO or pGlCDK2HA.NEO were monitored for growth every 6 h (Fig. 5A). The numbers of Giardia cells carrying pGlCDK1HA.NEO were similar to those of control cells carrying the vector plasmid. Interestingly, the numbers of Giardia cells expressing GlCDK2 significantly decreased compared to those of the control cells.

### Identification of GlCDK1- and/or GlCDK2-interacting cyclins in G. lamblia.

The activity of CDK is known to be modulated at the posttranscriptional level, that is, their phosphorylation status by CDK kinase or phosphatase, or through their association with other proteins, such as CDK inhibitors and cyclins. We searched GiardiaDB for candidate genes containing “cyclin” in their name and found that 9 of 16 had cyclin domain(s) (Table 1) but no deadbox, which is required for the ubiquitination-mediated degradation of cyclins in other organisms (29). Three of them (GL50803_3977, Glcyclin 3977; GL50803_14488, Glcyclin 14488; and GL50803_22394, Glcyclin 22394) have cyclin N (Pfam domain no. PF00134) and cyclin C (PF02984) domains, which are also present in cyclins of other organisms. These were annotated as cyclin B, cyclin A, and predicted protein, respectively. Four of the other Glcyclin candidates (GL50803_3095, Glcyclin 3095; GL50803_6584, Glcyclin 6584; GL50803_17505, Glcyclin 17505; and GL50803_93721, Glcyclin 93721) had a cyclin N domain (PF00134), whereas the other two (GL50803_13874, Glcyclin 13874; and GL50803_17400, Glcyclin 17400) had a different cyclin domain (PF08613) distinct from the cyclin N and cyclin C domains.

**TABLE 1:**
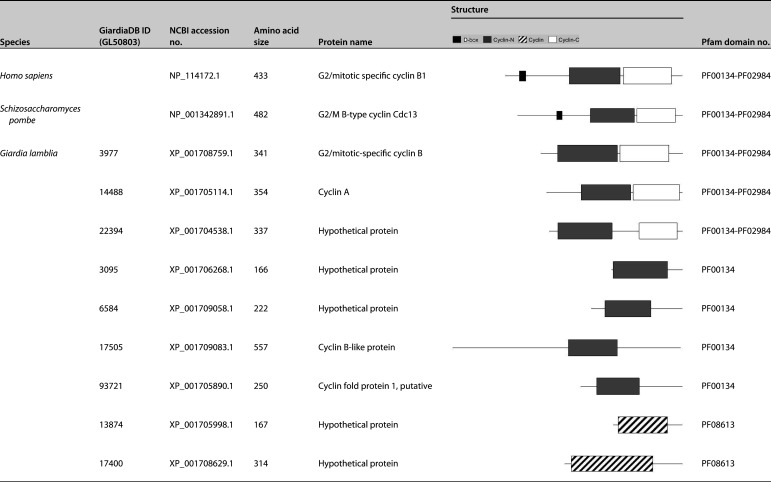
List of putative cyclins in GiardiaDB

**FIG 6:**
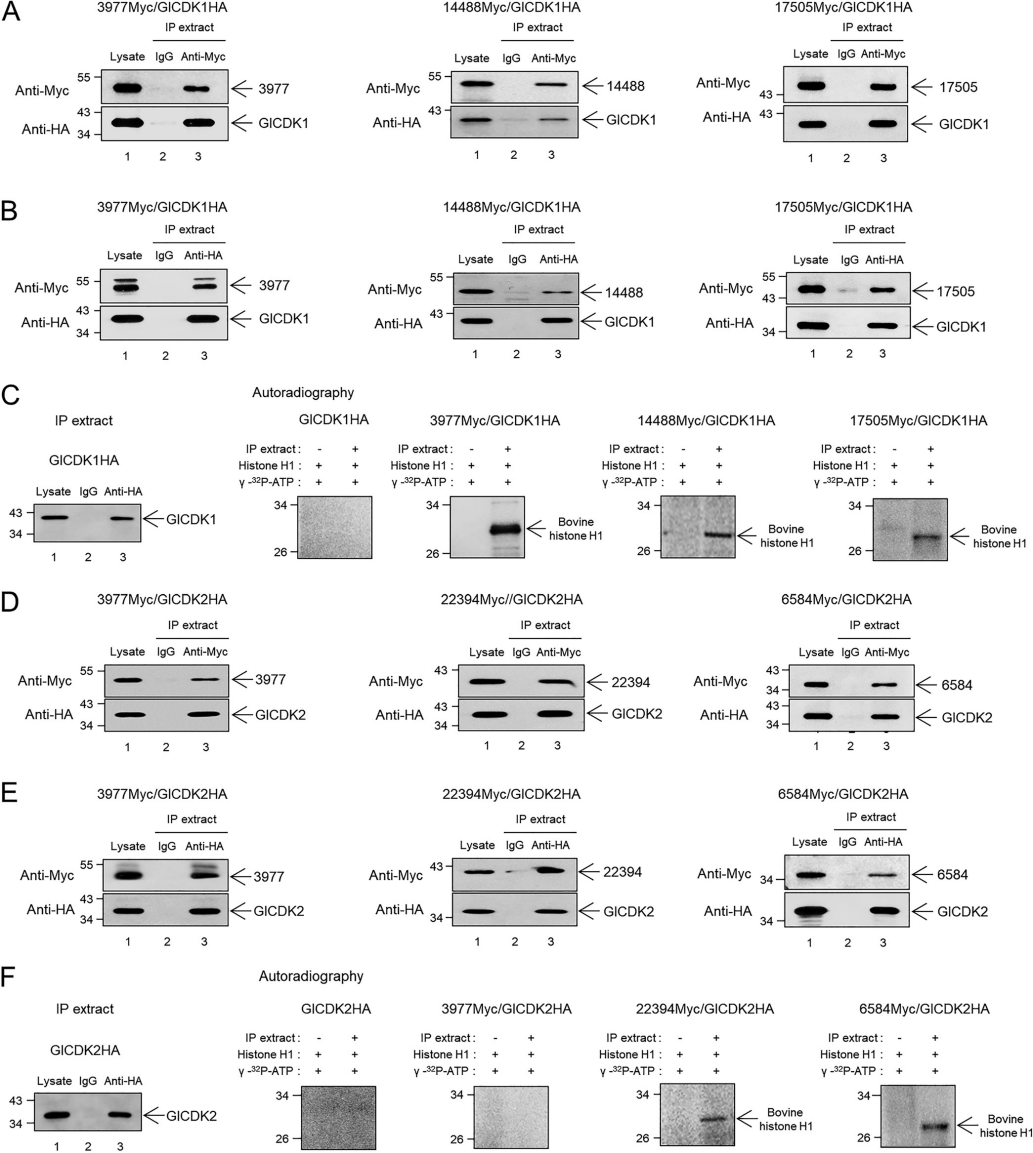
Coimmunoprecipitation (co-IP) of GlCDKs with Glcyclins and kinase activity of GlCDK-Glcyclin precipitates. (A to C) Co-IP of GlCDK1 with Glcyclins 3977, 14488, and 17505 and kinase activity of these GlCDK1-Glcyclin precipitates. (A and B) Giardia cells expressing HA-tagged GlCDK1 and the Myc-tagged Glcyclins were incubated with either (A) anti-Myc or (B) anti-HA IgG agarose, and the resulting precipitates (lane 3) were analyzed via Western blotting using anti-Myc or anti-HA antibodies. As controls, the same Giardia lysates were included without any treatment (lane 1) or with normal mouse IgG (lane 2). (C) These co-IP extracts were incubated with bovine histone H1 and γ-[^32^P]-ATP and then analyzed by autoradiography. Control cells only express HA-tagged GlCDK1. (D and E) Co-IP of GlCDK2 with Glcyclins 3977, 22394, and 6584 and kinase activity of these GlCDK2-Glcyclin precipitates. Giardia cells expressing HA-tagged GlCDK2 and one of the Myc-tagged Glcyclins, 3799, 22394, or 6584, were incubated either with (D) anti-Myc or (E) anti-HA IgG agarose. (F) These co-IP extracts were incubated with histone H1 and [γ-^32^P]-ATP and then analyzed by autoradiography. Control cells express only HA-tagged GlCDK2.

Plasmids expressing one of the nine putative Glcyclins in a Myc-tagged form were transfected into Giardia trophozoites expressing HA-tagged GlCDK1. Giardia lysates were prepared (lane 1), and precipitated with either control IgG (lane 2) or anti-Myc antibodies (lane 3) (Fig. 6A; see also Fig. S3A). These samples were analyzed by Western blotting using anti-Myc or anti-HA antibodies to examine whether Myc-tagged cyclins could co-IP with HA-tagged GlCDK1. Six of the nine putative cyclins did not co-IP with GlCDK1 (Fig. S3A). The other three cyclins (Glcyclins 3977, 14488, and 17505) coimmunoprecipitated with HA-tagged GlCDK1 with anti-Myc (Fig. 6A) or anti-HA antibodies (Fig. 6B). Next, these precipitates were examined for their ability to phosphorylate bovine histone H1 (Fig. 6C). The co-IP extracts prepared from Giardia cells expressing only HA-tagged GlCDK1 showed no kinase activity against bovine histone H1. Co-IP extracts prepared from Giardia expressing Glcyclin 3977, 14488, and 17505 with GlCDK1 demonstrated kinase activity against bovine histone H1.

These nine putative Glcyclins were also examined to determine whether they could be cognate partners of GlCDK2. Giardia cells expressing HA-tagged GlCDK2 and one of the nine cyclins tagged with Myc were precipitated using anti-Myc antibodies (Fig. 6D; see also Fig. S3B). Only Glcyclins 3977, 22394 and 6584 coimmunoprecipitated with HA-tagged GlCDK2 (Fig. 6E). The remaining six cyclins did not co-IP with GlCDK2 (Fig. S3B). In subsequent experiments, the three extracts were examined for their ability to phosphorylate bovine histone H1. Control extracts prepared from Giardia cells expressing only HA-tagged GlCDK2 did not exhibit any kinase activity (Fig. 6F), while co-IP extracts prepared from Giardia expressing HA-tagged GlCDK2 and Myc-tagged Glcyclin 22394 or 6584 demonstrated kinase activity toward bovine histone H1. Interestingly, co-IP extracts prepared from cells expressing GlCDK2 and Glcyclin 3977 did not phosphorylate bovine histone H1, despite their association. These results indicated that GlCDK1 functions with Glcyclins 3977, 14488, and 17505, whereas GlCDK2 plays a role with Glcyclins 22394 and 6584.

### Colocalization of GlCDKs with their cognate Glcyclins in Giardia cells.

**FIG 7:**
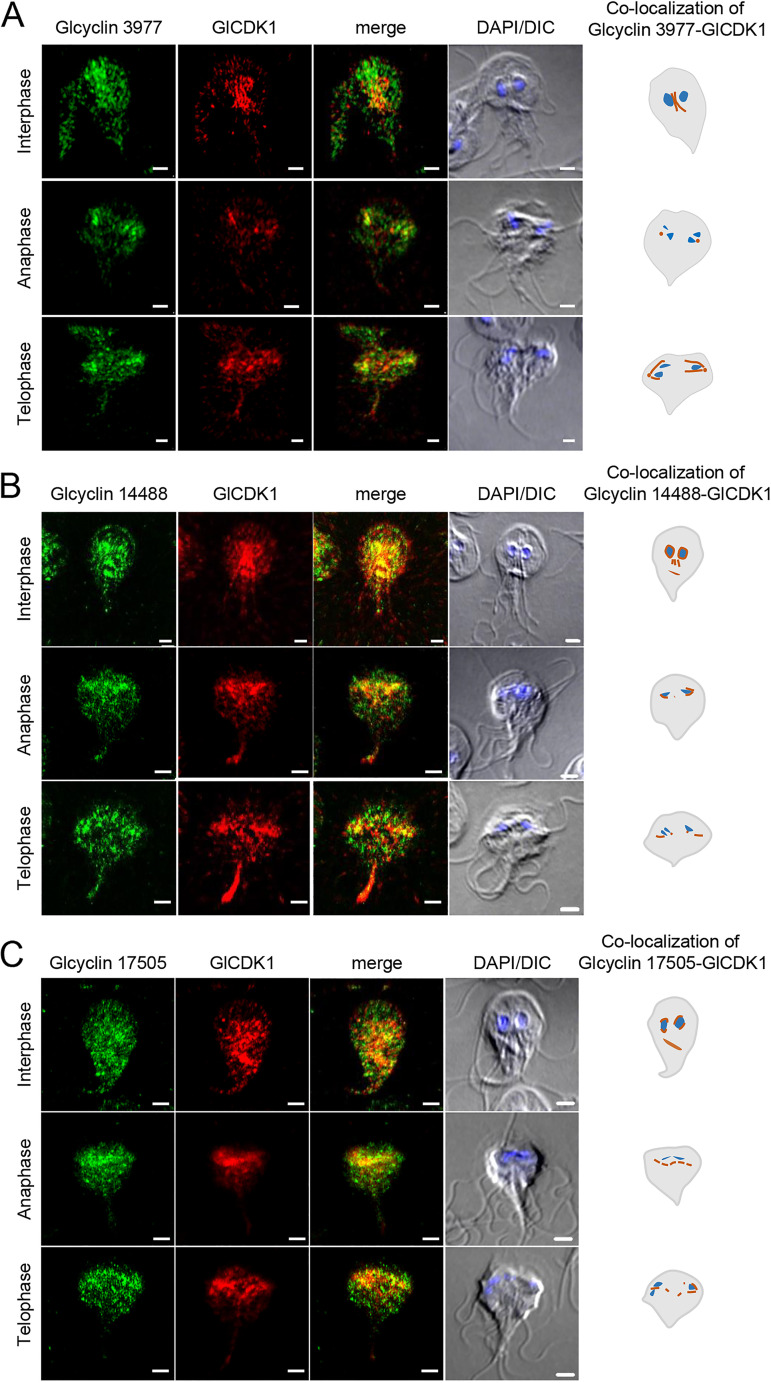

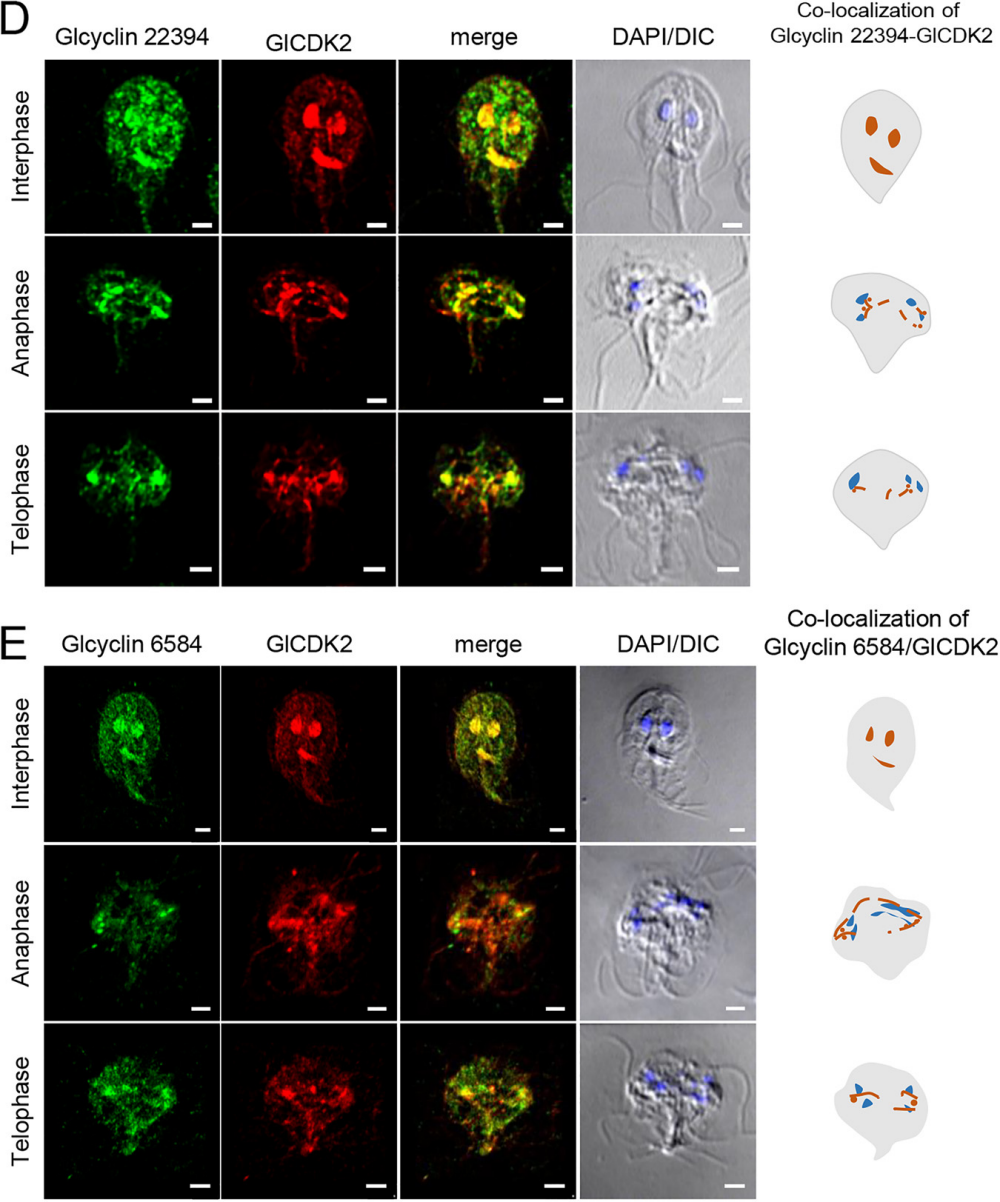
Colocalization of GlCDKs with their interacting Glcyclins in Giardia. (A) Localization of GlCDK1 and Glcyclin 3977 in Giardia expressing HA-tagged GlCDK1 and Myc-tagged Glcyclin 3977. (B) Localization of GlCDK1 and Glcyclin 14488 in Giardia expressing HA-tagged GlCDK1 and Myc-tagged Glcyclin 14488. (C) Localization of GlCDK1 and Glcyclin 17505 in Giardia expressing HA-tagged GlCDK1 and Myc-tagged Glcyclin 17505. (D) Localization of GlCDK2 and Glcyclin 22394 in Giardia expressing HA-tagged GlCDK2 and Myc-tagged Glcyclin 22384. (E) Localization of GlCDK2 and Glcyclin 6584 in Giardia expressing HA-tagged GlCDK2 and Myc-tagged Glcyclin 6584. IFAs of Giardia in interphase, anaphase, or telophase using anti-HA and anti-Myc antibodies. The cell morphology is represented by differential interference contrast (DIC) images. Scale bars = 2 μm.

Cells expressing HA-tagged GlCDK1 and Myc-tagged Glcyclin 3977 were analyzed by IFAs to determine whether these two components colocalized in interphase cells as well as in dividing cells (Fig. 7A). Double staining with anti-HA and anti-Myc antibodies indicated colocalization of CDK1 and Glcyclin 3977 at axonemes in interphase cells (*n* = 15), basal bodies during anaphase (*n* = 11), and basal bodies/mitotic spindle in cells in telophase (*n* = 9).

Staining of interphase Giardia cells expressing HA-tagged CDK1 and Myc-tagged Glcyclin 14488 with anti-HA and anti-Myc antibodies showed the colocalization of both proteins in the nuclear membrane, axoneme, and median body (*n* = 13) (Fig. 7B). Dividing cells at anaphase and telophase demonstrated labeling of basal bodies and mitotic spindles (*n* = 17 and 15, respectively).

Giardia cells carrying pGlCDK1HA.NEO and p17505Myc.PAC were costained with anti-HA and anti-Myc antibodies (Fig. 7C). In interphase Giardia cells, double staining revealed labeling of nuclear membrane and basal body (*n* = 15). GlCDK1 and Glcyclin 17505 were present in the mitotic spindles of the dividing Giardia at the anaphase (*n* = 13) and telophase (*n* = 17).

IFAs of cells coexpressing GlCDK2 and Glcyclin 22394 showed colocalization of these proteins at the nucleus and median body in interphase (*n* = 10), whereas both were found at basal bodies and mitotic spindles in dividing cells (*n* = 14) (Fig. 7D).

Double staining of Giardia cells coexpressing GlCDK2 and Glcyclin 6584 with anti-HA and anti-Myc antibodies showed that they colocalize in the nucleus and median body (interphase cells; *n* = 11) (Fig. 7E). In dividing cells, both Glcyclin 6584 and GlCDK2 were found in mitotic spindles and basal bodies (anaphase cells, *n* = 12; telophase cells, *n* = 12).

### Effect of GlCDK1-interacting Glcyclin knockdown on cell division in Giardia.

**FIG 8:**
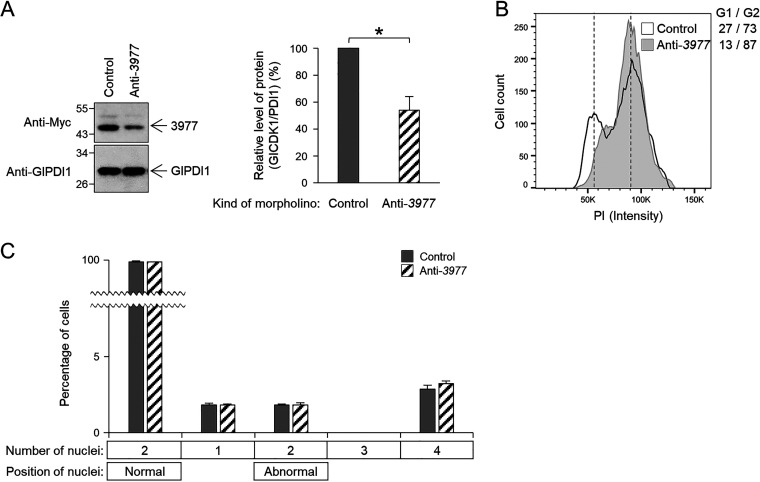
Morpholino-mediated knockdown of GlCDK1-interacting Glcyclin 3977. Giardia cells expressing Myc-tagged Glcyclin 3977 were prepared at 12 h after transfection with control (gray bars) or anti-*glcyclin* (hatched bars) morpholinos. (A) Western blots showing Glcyclin knockdown in Giardia. Bar graph of relative expression of Myc-tagged Glcyclin 3977 in cells treated with anti-*3977* morpholino compared with that in the control cells. (B and C) Effect of morpholino-mediated Glcyclin 3977 knockdown on (B) DNA content and (C) nuclear phenotypes of G. lamblia. Data are presented as the mean of three independent experiments. *, *P* < 0.05.

To determine the role of GlCDK1-interacting Glcyclins in the cell division of G. lamblia, we designed a Glcyclin-specific morpholino to block the expression of specific Glcyclins. A control morpholino was transfected into G. lamblia trophozoites expressing Myc-tagged Glcyclin by electroporation (Table S2). When the cells were harvested at 6 to 24 h posttransfection and analyzed for Glcyclin 3977 expression, the cells at 12 h posttransfection demonstrated maximal inhibition of Glcyclin 3977 expression to 54% (Fig. 8A). In Glcyclin 3977-depleted cells, the number of cells in the G2/M phase increased to 87%, compared with 73% of the control cells (Fig. 8B). Glcyclin 3977 knockdown did not affect the number of nuclei in Giardia trophozoites, and the percentage of cells undergoing cytokinesis did not change in Glcyclin 3977-depleted cells (Fig. 8C). Flow cytometric analysis to measure EdU incorporation in Giardia cells depleted of GlCDK1-interacting cyclins indicated that they were arrested at the G2/M phase. The levels of EdU incorporation were not affected in cells transfected with an anti-*3977* morpholino (Fig. S2C).

In contrast, only slight depletion of Glcyclin 14488 and 17505 was detected at 6 h posttransfection of an anti-*glcyclin* morpholino to 84 and 78%, respectively, as shown by Western blot analysis using anti-Myc antibodies (see Fig. S4A and D, respectively). Flow cytometry showed a slight increase (from 67% to 74%) or no change in the cell population at the G2/M phase in Glcyclin 14488- or 17505-depleted cells, respectively (Fig. S4B and E, respectively). At 6 h posttransfection, the cells did not show any statistically significant change in the nuclear phenotype (Fig. S4C and F, respectively). Flow cytometric analysis to measure EdU incorporation in Giardia cells depleted of GlCDK1-interacting cyclins indicated that they were arrested at the G2/M-phase, as expected. The degree of EdU incorporation was not affected in cells transfected with an anti-*14488* or anti-*17505* morpholino (Fig. S2D and E, respectively).

### Effect of GlCDK2-interacting Glcyclin knockdown on cell division in G. lamblia.

**FIG 9:**
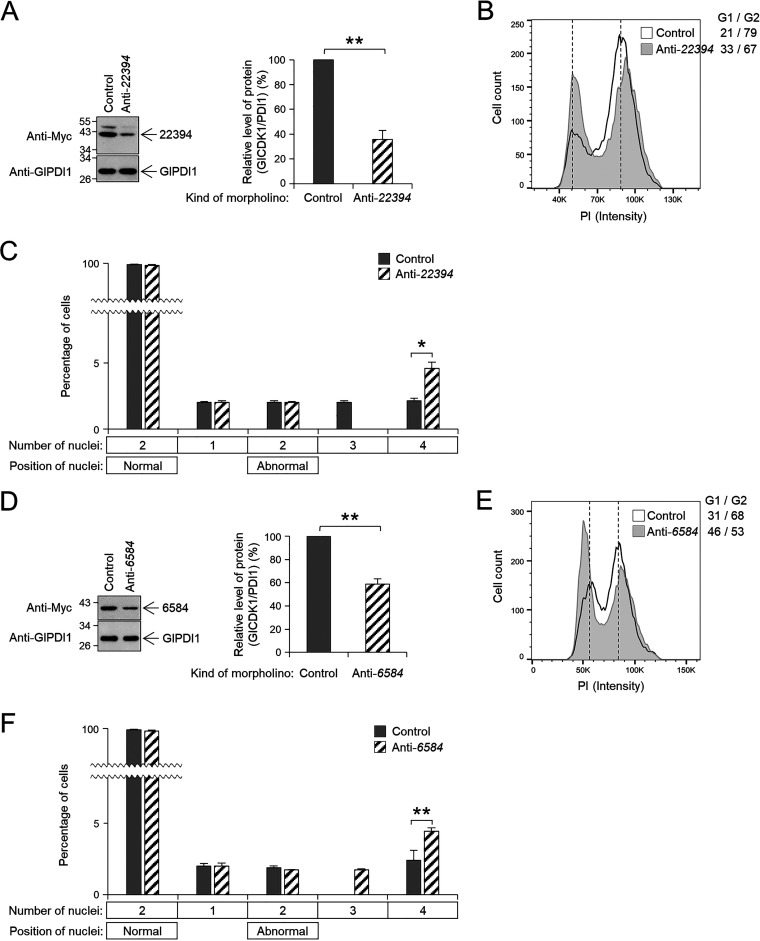
(A to F) Morpholino-mediated knockdown of GlCDK2-interacting Glcyclins 22394 (A to C) and 6584 (D to F). Giardia cells expressing Myc-tagged Glcyclins 22394 or 6584 were prepared at 6 h after transfection with control (gray bars) or anti-*glcyclin* (hatched bars) morpholino. (A and D) Western blots showing Glcyclin knockdown in Giardia. Bar graph of relative expression of Myc-tagged Glcyclins in cells treated with anti-*glcyclin* morpholinos compared with that in the control cells. (B, C, E, and F) Effect of morpholino-mediated Glcyclin 3977 knockdown on (B and E) DNA content and (C and F) nuclear phenotypes of G. lamblia. Data are presented as the mean of three independent experiments. *, *P* < 0.05; **, *P* < 0.01.

Transfection of an anti-*22394* morpholino into Giardia trophozoites resulted in a 64% depletion of Glcyclin 22394 compared to that of the cells treated with a control morpholino at 6 h posttransfection (Fig. 9A). Analysis of the DNA content of Glcyclin 22394-depleted cells indicated a significant increase in G1/S-phase cells from 21% to 33% (Fig. 9B). The percentages of cells with two normally positioned nuclei, cells with one nucleus, or two nuclei in an abnormal position were not changed in the cells transfected with an anti-*22394* morpholino (Fig. 9C). The percentage of cells undergoing cytokinesis was significantly increased to 4.4% from 1.1% of the control cells. In contrast, the percentage of cells with condensed DNA was not significantly increased. Glcyclin 22394-depleted cells demonstrated levels of EdU incorporation comparable to those of control cells (Fig. S2F).

Treatment of Giardia with an anti-*6584* morpholino for 6 h decreased the level of Glcyclin 6584 to 59% of that in the control cells (Fig. 9D). Flow cytometry showed an increase (from 31% to 46%) in cells at the G1/S phase in Glcyclin 6584-depleted cells (Fig. 9E). Decreased expression of Glcyclin 6584 did not cause a significant change in the percentage of cells with two normally positioned nuclei, cells with one nucleus, or cells with two nuclei in an abnormal position (Fig. 9F). In contrast, Glcyclin 6584 depletion resulted in a significant change in the percentage of cells undergoing cytokinesis to 4.8% compared with 1.6% of the control cells. EdU incorporation was not affected by Glcyclin 6584-knockdown (Fig. S2G).

### Effect of Glcyclin overexpression on Giardia growth.

Next, we determined whether the overexpression of these cyclins affected multiplication of G. lamblia. Giardia cells carrying the plasmid expressing Glcyclins 3977, 14488, 17505, 22394, or 6584 were monitored for growth every 6 h (Fig. 5B). Cell numbers of Giardia carrying p3977Myc.PAC or p14488Myc.PAC were slightly decreased compared to that of control cells carrying the empty vector. Giardia cells expressing either of the two GlCDK2-interacting Glcyclins (Glcyclins 22394 or 6584) or a GlCDK1-interacting Glcyclin (Glcyclin 17505) demonstrated significant defects of multiplication.

### Role of GlCDKs and Glcyclins in flagellum formation in G. lamblia.

**FIG 10:**
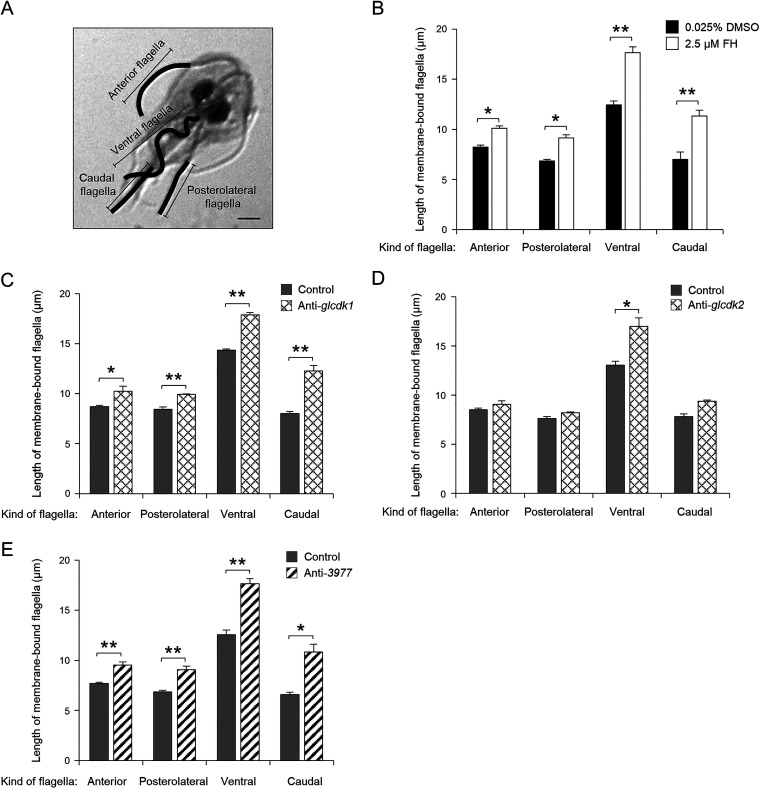
Effects of the CDK inhibitor FH and depletion of GlCDK1, GlCDK2, and Glcyclin 3977 on flagellar formation in Giardia. (A) Representative figure to show measurement of the membrane-bound anterior, posterolateral, ventral, and caudal flagella. Scale bar = 2 μm. (B) Effect of FH on flagellar length of Giardia. Cells were treated with 2.5 μM FH (open bars) or 0.025% DMSO (closed bars) for 3 h. (C to E) Effects of knockdown of GlCDK1, GlCDK2, and GlCDK1-interacting Glcyclin 3977 on flagellar length. Giardia cells were transfected with anti-*glcdk1* (C), anti-*glcdk2* (D), or anti-*3977* (E) morpholinos at a final concentration of 100 μM. The cells were examined for flagellar length at 12 h (for C and E) or 6 h (for D) posttransfection. The length of flagella was measured in 35 to 40 cells per condition. Data are presented as the mean of three independent experiments. *, *P* < 0.05; **, *P* < 0.01.

We investigated whether FH treatment modulates the process of flagellum formation in G. lamblia by measuring the length of all four pairs of flagella (Fig. 10A). Under control conditions, DMSO treatment for 3 h, the membrane-bound portion of ventral flagella was the longest. The remaining flagella exhibited shorter membrane-bound regions ranging from 6.9 to 8.0 μm (Fig. 10B). The cells treated with 2.5 μM FH showed a pronounced extension of all four flagella in their membrane-bound parts (ventral, 18 μm; caudal, 11 μm; posterolateral, 9.1 μm; anterior, 10 μm), suggesting that GlCDK is involved in the flagellum biogenesis.

GlCDK1-depleted cells produced an obvious extension in the length of all four pairs of flagella, with more distinct changes in the ventral and caudal flagella (Fig. 10C). In contrast, cells at 6 h posttransfection with an anti-*glcdk2* morpholino did not affect the Giardia flagella, except for the ventral flagella (Fig. 10D). Glcyclin 3977-depleted cells showed significant extension of all four pairs of flagella compared to that in Giardia treated with the control morpholino (Fig. 10E).

Giardia cells treated with the anti-*14488* or anti-*17505* morpholino for 6 h did not show any alteration in the length of four pairs of flagella (see Fig. S5A and B, respectively). Except for a slight increase in the length of the ventral flagella, the lengths of the other three flagella were not affected in anti-*22394* morpholino-treated cells (Fig. S5C). In addition, the length of the flagella was not altered in Giardia treated with the anti-*6584* morpholino (Fig. S5D).

Altogether, our results indicate that Glcyclin 6584 and 22394 play an important role in the whole process of cell division along with GlCDK2 in G. lamblia. However, this study did not provide evidence for their involvement in DNA synthesis. Conversely, GlCDK1 functions at a later stage of cell division. Interestingly, GlCDK1-Glcyclin 3977 plays a critical role in flagellar biogenesis.

## DISCUSSION

Unlike mitotic kinases, such as AK and PLK, CDKs are multifunctional kinases that play a role in the G1/S and G2/M phases of the mammalian cell cycle (18). The role of CDK in the Giardia cell cycle was initially examined using FH, a CDK-specific inhibitor (Fig. 1 and Fig. S1). The cell cycle was transiently arrested at the G1/S phase with a lower concentration of FH; however, the majority of cells were found in the G2/M phase when they were exposed to higher concentrations of FH for a longer duration. These results indicate that GlCDKs function in the G1/S and G2/M phases of the Giardia cell cycle. Specifically, FH-mediated GlCDK inhibition resulted in an increase of cells arrested at the prophase of mitosis, cells undergoing cytokinesis (Fig. 1D), and abnormally enlarged cells (Fig. 1E). A previous study reported that inhibition of GlCDK1-interacting Glcyclin 3977 caused an increase in the size of Giardia cells (8). However, the EdU incorporation experiment revealed that FH treatment did not affect DNA synthesis in G. lamblia, a key event in the G1/S phase (Fig. 1F).

We initially performed small experiments using another broad-spectrum inhibitor, Dinaciclib, and one CDK2-specific inhibitor, K03861, finding that FH was more efficient in arresting Giardia cells at the G2/M phase than the other two inhibitors. The active concentration of FH to arrest Giardia cells at the G2/M phase was 2.5 μM, which was higher than that of human carcinoma cells (200 nM) (30). In addition, the arrest of Giardia cells in the G2/M phase was reversed when FH was removed, suggesting that it could be used as a reagent to synchronize Giardia cells (Fig. 1C). Through morpholino-based specific depletion of GlCDK1 or GlCDK2, differentiation of their function was successfully achieved (Fig. 2, 3, and 10). Excavation of CDK inhibitors acting on only one of the two GlCDKs in future studies will aid in deepening our knowledge of GlCDKs and Glcyclins.

Characterization of the phenotypes caused by GlCDK-inhibition and GlCDK- or Glcyclin-depletion was mainly performed on the fixed Giardia cells via microscopic examinations and flow cytometric analyses (Fig. 1, 2, 3, 8, 9, and 10; Fig. S1, S4, and S5). To understand the GlCDK- and Glcyclin-mediated processes occurring in Giardia *in vivo*, observations of the live cells are essential to validate the results obtained from the experiments on the fixed cells. Live imaging of Giardia cells had been successfully performed in elaborate studies of biogenesis of the mitosome (31), flagellar length control (32), the role of never-in-mitosis A-related kinase 8455 (Nek8455) in cell division (33), identification of noncanonical actin-binding proteins (34), and involvement of the ventrolateral flange in Giardia attachment (35). The next step we should achieve is to establish the tool for live imaging of Giardia cells to confirm the data derived from the fixed cells.

In mammals, CDK activity is modulated at the posttranscriptional level through several mechanisms. First, a specific sequence of phosphorylations and dephosphorylations of inhibitory amino acids of CDK can activate or inhibit its activity (14, 36). CDK-activating kinases and CDK phosphatases play a role in this complex control, and their activity has been extensively characterized in yeast cells (37, 38). A search in the GiardiaDB indicated the presence of homologous proteins for membrane-associated fission yeast CDK Cdc2 inhibitory kinase (GL50803_15572) and dual-specificity phosphatase Cdc25 (GL50803_4369). Another level of control for CDK activity is driven by the association of CDK inhibitors, which serve as brakes to halt cell cycle progression under unfavorable conditions (16). No obvious homolog for the CDK inhibitors was found in the GiardiaDB. Most importantly, association with their cognate cyclins is essential for CDK activation. Cyclins are regulatory subunits that function as gears for CDKs, and a plethora of information is available in a diverse array of eukaryotic cells (15). In this study, five Glcyclins (3977, 14488, 17505, 22394, and 6584) were associated with GlCDKs through co-IP/kinase assays (Fig. 6 and Fig. S3).

Interestingly, these cyclins found in Giardia do not have the deadbox required for proteasome-mediated degradation, which may be related to the absence of the APC components, except for APC11 (GL50803_8432) in G. lamblia (7, 8, 39). Instead of proteasome-mediated degradation, the Giardia cell cycle machinery is modulated via another type of control system, and regulation by phosphorylation has been proven for Glcyclin 3977 using kinase and phosphatase inhibitors (8). Recently, protein small ubiquitin-like modifier (SUMO)ylation was reported to be involved in diverse cell cycle-related processes in Giardia (40). Genes encoding this pathway are conserved in Giardia (41, 42), and SUMOylation of arginine deiminase has been found to be involved in the encystation of Giardia (43, 44). Use of the GPS-SUMO 1.0 tool predicted potential sites of SUMOylation in Glcyclins 3977, 14488, 17505, and 22394 (data not shown). Therefore, it is possible that the protein levels of Glcyclins fluctuate due to SUMOylation.

The transcript levels of *glcdk* and *glcyclin* genes were also obtained from the transcriptome sequencing (RNA-seq) analysis (6) and quantitative real-time PCR (unpublished result, J Kim and S-J Park) of Giardia cells enriched in G1/S and G2/M phases. The level of *glcdk2* transcript was significantly increased at the G2/M phase (2- to 4-fold). The transcript of *glcyclin 13874* increased in Giardia cells at the G1/S phase (1.5-fold). Interestingly, dual RNA sequencing analysis of Giardia interacting with human intestinal epithelial cells indicated that expression of 2 GlCDKs and 11 Glcyclins was decreased (45). The exact mechanisms of how the number of Glcyclins is varied and which Glcyclins exert their differential function in the Giardia cell cycle are important issues that need to be addressed in the future.

The number of CDKs varies depending on the organism. Single CDKs with multiple functions have been reported in budding and fission yeasts, whereas more than 15 CDKs with more specialized but highly redundant functions in cell cycle, motility, and transcription control have been found in humans (18). In G. lamblia, only two CDKs, GlCDK1 and GlCDK2, are expected to bind to cyclins (46). Our results showed that morpholino-mediated depletion of GlCDK1 caused a moderate increase in cells in the G2/M phase (Fig. 2B) and in cells undergoing cytokinesis (Fig. 2C) but no alteration in EdU incorporation (see Fig. S2A). On the other hand, GlCDK2 depletion resulted in cell cycle arrest at an earlier point in the cell cycle, the G1/S phase (Fig. 3B); therefore, it exerted a more dramatic effect on the nuclear phenotype and cytokinesis (Fig. 3C). Consistent with the results using FH (Fig. 1F), GlCDK2-depletion did not cause any change in the degree of EdU incorporation (Fig. S2B), indicating that GlCDK2 is involved in the overall process of the Giardia cell cycle; however, its role in DNA synthesis has not been proven.

In mammals, CDK2/CycE is a major cell cycle component that controls the G1/S/G2 phases. In addition, CDK2/CycA regulates the phosphorylation of transcription factors (47, 48). One of the important roles of some CDKs is the reversible phosphorylation of the C-terminal domain of RNA polymerase II, resulting in transcriptional control (49, 50). Comparative genomic investigation of CDKs indicates that cell cycle-related CDKs are present, but no clear orthologues of transcription-related CDKs are present in *Trypanosoma* and Giardia (51). In G. lamblia, GlCDK2 has been reported to phosphorylate Myb2, a well-known transcription factor whose expression is induced during encystation (19). Limited information is available about the role of GlCDKs in differentiation of G. lamblia. Expression analyses of the encystation process at various time points indicated that the transcripts of *glcdk2* and *glcyclin 22394* increased at a later stage of encystation (52). In addition, expression of two putative Glcyclins, Glcyclin 15532 and 2661 was upregulated at early and late time points of encystation, respectively. Most of all, several experiments should be performed to examine whether GlCDK2 interacts with Glcyclins 15532 or 2661. If that is the case, mining of the transcription machinery controlled by GlCDK2 and these Glcyclins and their physiological implication with respect to differentiation of Giardia are interesting topics for future investigation.

GlCDK1 was found to be associated with three Glcyclins: 3977, 14488, and 17505 (Fig. 6A and B). The resulting co-IP extracts of GlCDK1 with these Glcyclins clearly demonstrated phosphorylation activity against bovine histone H1. The association of GlCDK1 with Glcyclin 3977 and the kinase activity of these extracts have been previously reported (8). For the other two Glcyclins that precipitated with GlCDK1, it is too early to draw any conclusion regarding their function in the Giardia cell cycle because the inhibition of their expression via the anti-*glcyclin 14488* or *17505* morpholino was minimal or absent (Fig. S4). However, there is still a possibility that these Glcyclins are involved in Giardia cell cycle control because they were found at mitotic spindles of dividing cells (Fig. 7B and C).

Glcyclins, 3977, 22394, and 6584 were associated with GlCDK2 (Fig. 6D and E). Interestingly, only co-IP extracts of GlCDK2 with Glcyclin 22394 or 6584 demonstrated kinase activity toward bovine histone H1. The phenotype of GlCDK2-knockdown cells (Fig. 3) correlated with the defects caused by the morpholino-mediated knockdown of Glcyclin 22394 and 6584 (Fig. 9), suggesting that both are cognate cyclins of GlCDK2.

Our study provides some clues regarding the function of the two GlCDKs and plausible cyclin(s) for each GlCDK. GlCDK1 plays a role in the later stages of the Giardia cell cycle, along with Glcyclin 3977, whereas GlCDK2 is the main kinase acting in the earlier phases in conjunction with Glcyclins 22394 and 6584. However, these CDKs may not play exclusive and distinct roles. Rather, it is likely that these GlCDKs still play redundant functions and share Glcyclins. For example, although the co-IP extracts of GlCDK2-Glcyclin 3977 did not show any kinase activity for bovine histone H1 (Fig. 6E), it is still possible that this complex plays a role in cell cycle control or that the function of this complex could be revealed via alternative assays.

Although bovine histone H1 was used as a substrate to measure kinase activity of GlCDK-Glcyclin complexes of G. lamblia, this protozoan lacks histone H1, whereas it retains other histones such as histones H2a, H2b, H3, and H4 (53). Numerous cell cycle-related proteins had been reported as substrates of human CDK2 (54) and CDKs of the fission yeasts (55). Orthologs of these proteins found in GiardiaDB could be used as a genuine substrate of GlCDKs, which includes GlPLK (GL50803_104150), GlCdc25 (dual-specificity phosphatase; GL50803_9073), WEE kinase (GL50803_115572), GlSMC4 (chromosome condensation protein; GL50803_17465), GlOrc1 (DNA replication origin-binding protein: GL50803_17103), and GlFen1 (Flap structure-specific endonuclease; GL50803_16953).

Lastly, the role of GlCDKs in flagellum formation was confirmed using FH (Fig. 10B). GlCDK1 plays a key role in this process, as demonstrated in morpholino-mediated knockdown experiments with GlCDKs (Fig. 10C). During interphase in Giardia cells, flagellar length should be homeostatically maintained. In contrast, flagellar biogenesis in Giardia is an essential process coupled with cytokinesis, in which a flagellum-driven force is required instead of typical abscission via the actin-myosin ring (2). A previous study demonstrated that overexpression of a dominant negative mutant of the motor protein kinesin-13 resulted in Giardia with longer flagella (56). Depletion of Nek8445 results in flagellar shortening in Giardia (33). It has been suggested that GlPLK regulates flagellar length through phosphorylation of Giardia kinesin-13 (57). Among the five Glcyclins, Giardia cells treated with an anti-*3977* morpholino showed a distinct extension of the flagella (Fig. 10E). Thus, our study added GlCDK1-Glcyclin 3977 to the list of components involved in flagellar length regulation.

## MATERIALS AND METHODS

### Cultivation of Giardia.

Giardia lamblia trophozoites (strain WB, ATCC 30957) were grown in modified TYI-S-33 medium (2% casein digest, 1% yeast extract, 1% glucose, 0.2% NaCl, 0.2% l-cysteine, 0.02% ascorbic acid, 0.2% K_2_HPO_4_, 0.06% KH_2_PO_4_, pH 7.1) supplemented with 10% heat-inactivated calf serum (Sigma-Aldrich) and 0.5 mg/mL bovine bile at 37°C (58). The cells were grown in 15-mL tubes with a screw cap with 5% CO_2_ to 80% confluence (approximately 72 h) before 50 μL of the culture was transferred into 10 mL of fresh medium. Transfected Giardia strains were cultured in TYI-S-33 medium containing the appropriate antibiotics at the following concentrations: 50 μg/mL puromycin, 600 μg/mL G418, and 75 μg/mL blasticidin.

Giardia trophozoites (1 × 10^5^ cells/mL) were cultured in modified TYI-S-33 medium to 70 to 80% confluence. A portion of these cells were treated with 100 nM nocodazole (Sigma-Aldrich) for 2 h and harvested as G2/M-phase cells.

### Inhibitor treatment of Giardia trophozoites.

Giardia cells (1 × 10^6^ cells/mL) were treated with the CDK inhibitors (flavopiridol-HCl [FH; Cayman Chemical], K03861 [Selleckchem], and dinaciclib [Selleckchem]) at various concentrations (1.0 to 10.0 μM) for 24 h before flow cytometry analysis. In the following experiment, Giardia cells were incubated with 2.5 μM FH for various time periods, from 1 h. Control cells were treated with 0.025% DMSO for 1 to 24 h.

Another set of Giardia cells was incubated with lower concentrations of FH (10 to 1,000 nM) for 1 h. In subsequent assays, Giardia cells were exposed to 50 nM FH for 30 min to 3 h, while control cells were incubated with 0.0005% DMSO for the corresponding time periods. Data are presented as representative histograms of three independent experiments.

### Flow cytometry.

Both FH- and DMSO-treated G. lamblia cells were analyzed for DNA content using flow cytometry as previously described (59). Briefly, harvested cells were resuspended in 50 μL of TYI-S-33 culture medium and treated with 150 μL of cell fixative (1% Triton X-100, 40 mM citric acid, 20 mM Na_2_HPO_4_, 200 mM sucrose, pH 3.0) for 5 min at room temperature. The cells were diluted with 350 μL of diluent buffer (125 mM MgCl_2_ in phosphate-buffered saline [PBS; 137 mM NaCl, 2.7 mM KCl, 10.1 mM Na_2_HPO_4_, and 2 mM KH_2_PO_4_, pH 7.4]) and then incubated with 10 μg/mL propidium iodide (PI; Sigma-Aldrich) and 2.5 μg RNase A (Sigma-Aldrich) at 37°C for 30 min. The results were analyzed using FlowJo software (ver. 20). Data are presented as representative histograms of three independent experiments.

### Microscopic observation of Giemsa-stained cells.

Giardia trophozoites (1 × 10^6^ cells/mL) were treated with 2.5 μM FH for 3 h or 50 nM FH for 1.5 h and stained with Giemsa. Control cells were treated with 0.025% or 0.0005% DMSO for 3 or 1.5 h, respectively. As previously described (13), cells attached to the slides were fixed with 100% methanol for 10 min. After staining with 10% Giemsa, cells were washed with distilled water and observed under a microscope (Axiovert 200; Carl Zeiss). At least 1,000 cells were examined to score their nuclear phenotypes, including position and number. The data are presented as the mean ± standard deviation (SD) of three independent experiments.

To measure the length of flagella, Giemsa-stained cells were observed under a microscope, and their differential interference contrast images were analyzed using Fiji (60). The membrane-bound regions of the four types of flagella were analyzed using the line freehand tracing mode in ImageJ (http://imagej.nih.gov/ij/). The flagellar length of 35 to 40 cells was measured per experiment. The data were measured as the mean ± SD of three independent experiments.

### Size measurement of Giardia cells.

To determine the size distribution of FH- and DMSO-treated cells, cells were incubated with CellMask plasma membrane stains (C10046; Thermo Fisher Scientific) to mark the surface of the entire cell. The cells were mounted in ProLong antifade with 4,6-diamino-2-phenylindole (Invitrogen) and examined using an LSM700 confocal microscope (Carl Zeiss). After analysis using ZEN imaging software (Blue edition 2.1; Carl Zeiss), the size distribution of the analyzed cells was plotted as a histogram. This experiment was performed on three independently prepared cells, and a representative image is presented.

### 5-ethynyl-2′-deoxyuridine (EdU) incorporation assays and click chemistry reaction.

EdU was supplied by the EdU proliferation kit (Abcam), and PI was used to monitor Giardia cells during the cell cycle. EdU was used to monitor DNA synthesis during the S phase. Giardia trophozoites were seeded at a density of 1 × 10^5^ cells/mL. After 24 h, the medium was replaced with fresh medium containing the following components at the indicated concentrations and periods with 70 μM EdU under dark conditions: aphidicolin, 6 μM, 6 h; FH, 50 nM, 1.5 h; and FH, 2.5 μM, 3 h. The resulting cell pellets were resuspended in 50 μL of TYI-S-33 medium, and the cells were fixed and permeabilized with 150 μL of the cell fixative used for flow cytometry. The cells were washed with PBS, and EdU-labeled DNA was detected according to the manufacturer’s instructions. The cells were incubated with 500 μL of the reaction solution for 30 min at room temperature in the dark. After washing with PBS, the cells were incubated with 10 μg/mL PI and 2.5 μg/mL RNase A for 30 min at 37°C and subsequently analyzed using a flow cytometer. Representative histograms from three independent experiments are presented.

### Plasmids of the hemagglutinin (HA) epitope-tagged GlCDKs.

All plasmids and primers used in this study are listed in Tables S1 and S2, respectively. A 1,124-bp DNA fragment consisting of the 200-bp promoter region and *glcdk1* gene was obtained from Giardia genomic DNA by PCR using primers CDK1-F-NotI and CDK1-R-HindIII. The NotI and HindIII sites were used to clone the resulting DNA fragment into pKS-3HA.NEO (61) to produce pGlCDK1HA.NEO. The plasmid was verified by DNA sequencing (Macrogen, Seoul, South Korea).

A DNA fragment containing a 200-bp promoter region and an 876-bp ORF of *glcdk2* was obtained from Giardia genomic DNA using the primers CDK2-F-NotI and CDK2-R-HindIII and cloned into the NotI and HindIII sites of pKS-3HA.NEO to produce pGlCDK2HA.NEO.

### Transfection.

pGlCDK1HA.NEO or pGlCDK2HA.NEO (20 μg) was transfected into 1 × 10^7^ Giardia trophozoites by electroporation under the following conditions: 350 V, 1,000 μF, and 700 Ω (Bio-Rad). Transfected trophozoites were initially selected in a medium containing 150 μg/mL G418 before the selected cells were transferred into a medium containing 600 μg/mL G418. HA-tagged GlCDK expression was observed using Western blotting, and Giardia carrying pKS-3HA.NEO was used as the control. Six sets of transfections were performed for each experiment.

Plasmids expressing Myc-tagged Glcyclins were also transfected into Giardia trophozoites, as described above. Transfected trophozoites were initially selected in TYI-S-33 medium containing 10 μg/mL puromycin before the concentration of puromycin was increased to 50 μg/mL. Expression of Myc-tagged Glcyclins was examined by Western blotting using anti-Myc antibodies.

### Western blotting.

Lysates were prepared from Giardia cells carrying pKS-3HA.NEO, pGlCDK1HA.NEO, or pGlCDK2HA.NEO. The proteins (10 to 50 μg) were separated by sodium dodecyl sulfate-polyacrylamide gel electrophoresis (SDS-PAGE) and transferred onto a polyvinylidene fluoride membrane (Millipore) using a semidry transfer system. The membrane was incubated with mouse monoclonal anti-HA (1:1,000; Sigma-Aldrich) antibodies in blocking solution (50 mM Tris-HCl, 5% skim milk, and 0.05% Tween 20 in PBS) at 4°C overnight. The membranes were subsequently incubated with horseradish peroxidase-conjugated secondary antibodies, and immunoreactive proteins were visualized using an enhanced chemiluminescence system (Thermo Fisher Scientific). The membrane was treated with stripping buffer (Thermo Fisher Scientific) and incubated with G. lamblia protein disulfide isomerase 1 (GlPDI1; GL50803_29487) polyclonal rat antibodies (1:10,000) as a loading control (59).

### Immunofluorescence assay.

To increase the rate of cell division, Giardia cells grown for 72 h were incubated for 4 to 5 h with fresh medium, as previously described (62). The harvested trophozoites were fixed on a slide with PBS containing 2% paraformaldehyde, 100 μM 3-maleimidobenzoic acid *N*-hydroxysuccinimide ester (Sigma-Aldrich), and 100 μM ethylene glycol-bis(succinimidyl succinate) (Thermo Fisher Scientific) for 30 min at 37°C. The cells were then treated with 100 mM glycine in PBS for 5 min and permeabilized with 0.5% Triton X-100 in PBS for 10 min at room temperature. After three washes with PBS, the slides were blocked with 3% BSA in PBS for 30 min, incubated with primary antibodies, and then treated with secondary antibodies. After staining, the slides were mounted and observed under a Zeiss LSM700 confocal microscope (Carl Zeiss).

The following antibodies were used at the indicated dilutions: anti-HA rat monoclonal antibodies (clone 3F10, Roche Applied Science; 1:100), anti-acetylated-α-tubulin mouse antibodies (clone 6-11B-1, T7451, Sigma-Aldrich; 1:800), anti-Myc mouse antibodies (Thermo Fisher Scientific; 1:50), anti-Glcentrin mouse antibodies (59), Alexa Fluor 555-conjugated donkey anti-rat IgG (Molecular Probes; 1:100), and Alexa Fluor 488-conjugated donkey anti-mouse IgG (Molecular Probes; 1:100).

### Cell counting of Giardia growth.

Giardia trophozoites carrying the expression plasmid for GlCDKs and Glcyclins were inoculated at 1 × 10^4^ cells/mL, and cell numbers were determined every 6 h for up to 48 h using a hemocytometer. The data were derived from three independent experiments.

### Morpholino-mediated knockdown experiment.

The expression of GlCDKs or Glcyclins was blocked using a morpholino, as previously described (63). Specific morpholinos for GlCDK1, GlCDK2, and five Glcyclins were designed using Gene Tools, and their sequences are listed in Table S2. Nonspecific sequences were used as the control morpholino. Each morpholino (100 μM) was transfected into Giardia cells (5 × 10^6^) via electroporation. Cells grown for various durations from 6 to 24 h were prepared for Western blotting using anti-HA or anti-Myc antibodies to measure GlCDKs or Glcyclins levels, respectively. After 6 or 12 h of transfection, the flagellar length and nuclear phenotypes, including the position and number of nuclei and the number of dividing cells were determined as described above. Additionally, cells transfected with control, anti-*glcdks*, or anti-*glcyclins* morpholinos were harvested after 6 to 24 h, and their DNA content was analyzed using flow cytometry and EdU assay. Transfection was performed at least four times, and the data presented were derived from three independent knockdown experiments.

### Construction of the Myc epitope-tagged Glcyclins.

Nine putative cyclins from the GiardiaDB were expressed in Myc-tagged forms in Giardia trophozoites, which were then used for co-IP experiments with HA-tagged GlCDK1 or GlCDK2. DNA fragments of the putative *glcyclin* genes were obtained from Giardia genomic DNA via PCR using the primers listed in Table S2. NotI and ClaI sites were used to clone DNA encoding Glcyclin 14488, 22394, 3095, 6584, 17505, 93721, and 13874 into pKS-3Myc.PAC (61) to obtain the corresponding Glcyclin-expressing plasmids. For Glcyclins 3977 and 17400, the XbaI site was used instead of ClaI to construct the expression plasmids. The inserted fragments were confirmed by DNA sequencing (Macrogen).

### Co-IP assays.

Giardia cells carrying one of the two CDK plasmids (pGlCDK1HA.NEO and pGlCDK2HA.NEO) and one of the nine cyclin plasmids were harvested, washed twice with ice-cold PBS, and lysed in protein lysis buffer (10 mM Tris-HCl, 5 mM EDTA, 130 mM NaCl, and 1% Triton X-100, pH 7.4) containing a protease inhibitor cocktail (GenDEPOT). Lysates were precleared with protein A/G agarose (Thermo Fisher Scientific) for 1 h at 4°C. Subsequently, precleared lysates were incubated with monoclonal mouse anti-HA agarose beads (Sigma-Aldrich) or monoclonal mouse anti-Myc antibodies (Clontech) at 4°C overnight. The beads were washed twice with co-IP washing buffer (50 mM Tris-HCl, 150 mM NaCl, and 1% Triton X-100, pH 7.4) for 10 min and resuspended in 2 × SDS sample buffer. The precipitates were then analyzed by Western blotting using anti-HA or anti-Myc antibodies.

### Histone kinase assays.

For Giardia cells showing the coprecipitation of GlCDK and Glcyclin, co-IP extracts were prepared using anti-Myc antibodies as described above and then used for histone kinase assays. These extracts were incubated with 5 μg bovine histone H1 and 2 μCi [γ-^32^P] ATP together with 25 μM cold ATP for 20 min at 25°C in kinase assay buffer containing 150 mM NaCl, 25 mM HEPES, pH 7.5, 10 mM Mg^2+^, and 1 mM dithiothreitol (DTT). Reactions were stopped by adding SDS sample buffer and boiling. The samples were analyzed by SDS-PAGE, followed by autoradiography. As controls, extracts of Giardia cells carrying pGlCDK1HA.NEO or pGlCDK2HA.NEO were immunoprecipitated with anti-IgG or anti-HA antibodies and then used for histone kinase assays.

### Statistical analyses.

Data are presented as the mean ± SD of three independent experiments. Statistical analyses for pairwise comparisons were performed using Student’s *t* test (SigmaPlot ver. 9; Systat Software, Inc.). Differences were considered statistically significant at *P* values of <0.05.
