# Supplementary material for: Functional Differentiation of Cyclins and Cyclin-Dependent Kinases in Giardia lamblia
Source: Microbiol Spectr. 2023 Mar 6;11(2):e04919-22. doi: 10.1128/spectrum.04919-22 (PMC10100927; doi:10.1128/spectrum.04919-22)
Supplement: Supplemental file 1 — Supplemental material. Download spectrum.04919-22-s0001.pdf, PDF file, 1.0 MB [file spectrum.04919-22-s0001.pdf]

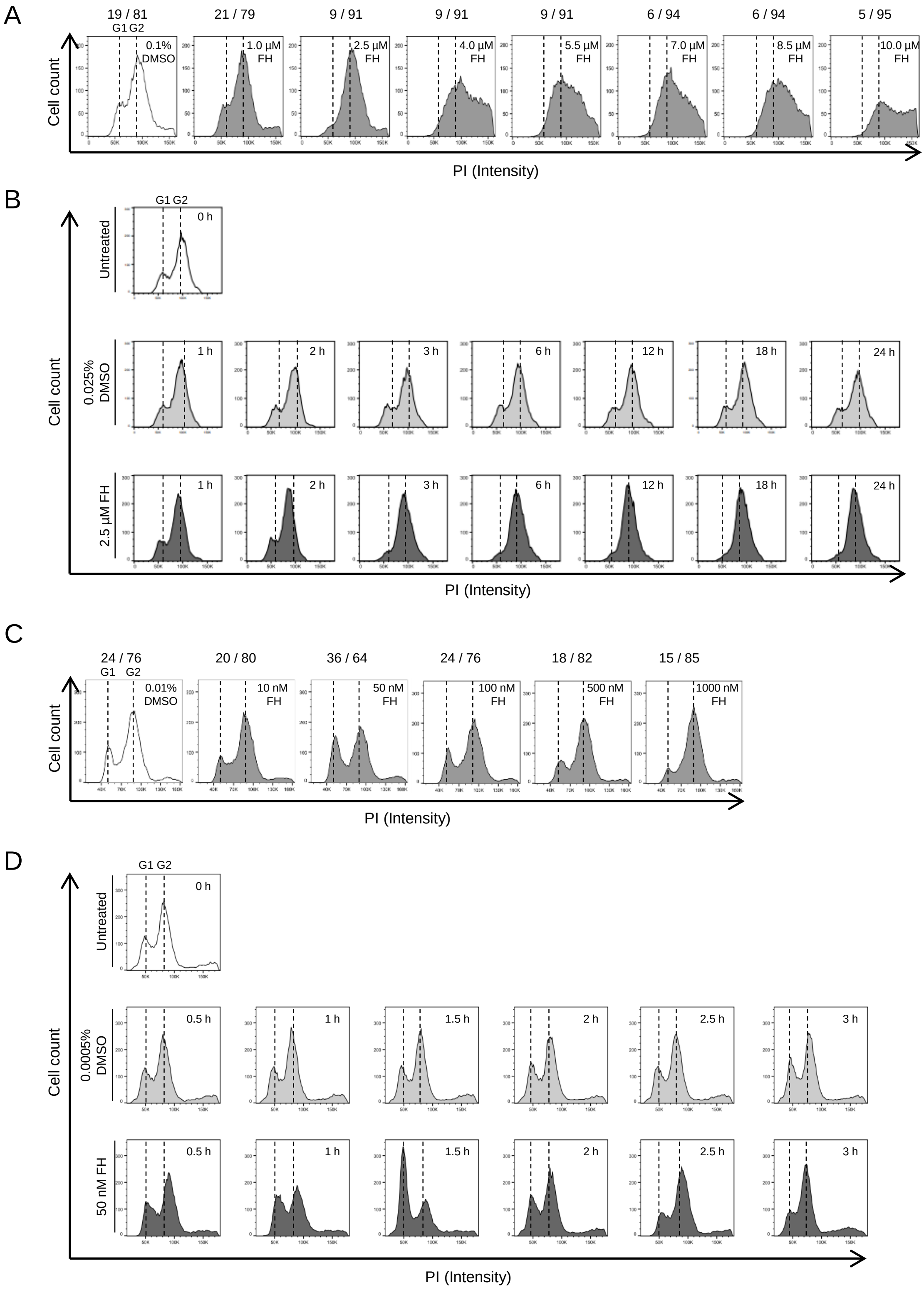
A
19 / 81
21 / 79
9 / 91
9 / 91
9 / 91
6 / 94
6 / 94
5 / 95
G1 G2
0.1% DMSO
1.0 µM FH
2.5 µM FH
4.0 µM FH
5.5 µM FH
7.0 µM FH
8.5 µM FH
10.0 µM FH
Cell count
PI (Intensity)
B
G1 G2
0 h
Untreated
0.025% DMSO
2.5 µM FH
1 h
2 h
3 h
6 h
12 h
18 h
24 h
Cell count
PI (Intensity)
C
24 / 76
20 / 80
36 / 64
24 / 76
18 / 82
15 / 85
G1 G2
0.01% DMSO
10 nM FH
50 nM FH
100 nM FH
500 nM FH
1000 nM FH
Cell count
PI (Intensity)
D
G1 G2
0 h
Untreated
0.0005% DMSO
50 nM FH
0.5 h
1 h
1.5 h
2 h
2.5 h
3 h
Cell count
PI (Intensity)

FIG S1 Effect of the cyclin-dependent kinase (CDK) inhibitor, flavopiridol-HCl (FH), on the DNA content of *Giardia*. (A) *Giardia* was treated with varying concentrations of FH (1.0–10.0 μM) for 24 h. DNA content was measured using flow cytometry. Control cells were incubated with 0.1% dimethyl sulfoxide (DMSO). (B) *Giardia* cells were incubated with 2.5 μM FH for varying durations, ranging from 1–24 h. Control cells were treated with 0.025% DMSO for 1–24 h. (C) *Giardia* cells were incubated with lower concentrations of FH (10–1000 nM) for 1 h and analysed by flow cytometry. Control cells were treated with 0.01% DMSO. (D) *Giardia* cells were exposed to 50 nM FH for 30 min–3 h, while control cells were incubated with 0.0005% DMSO for the corresponding time points. Data shown are representative for three independent replicates.

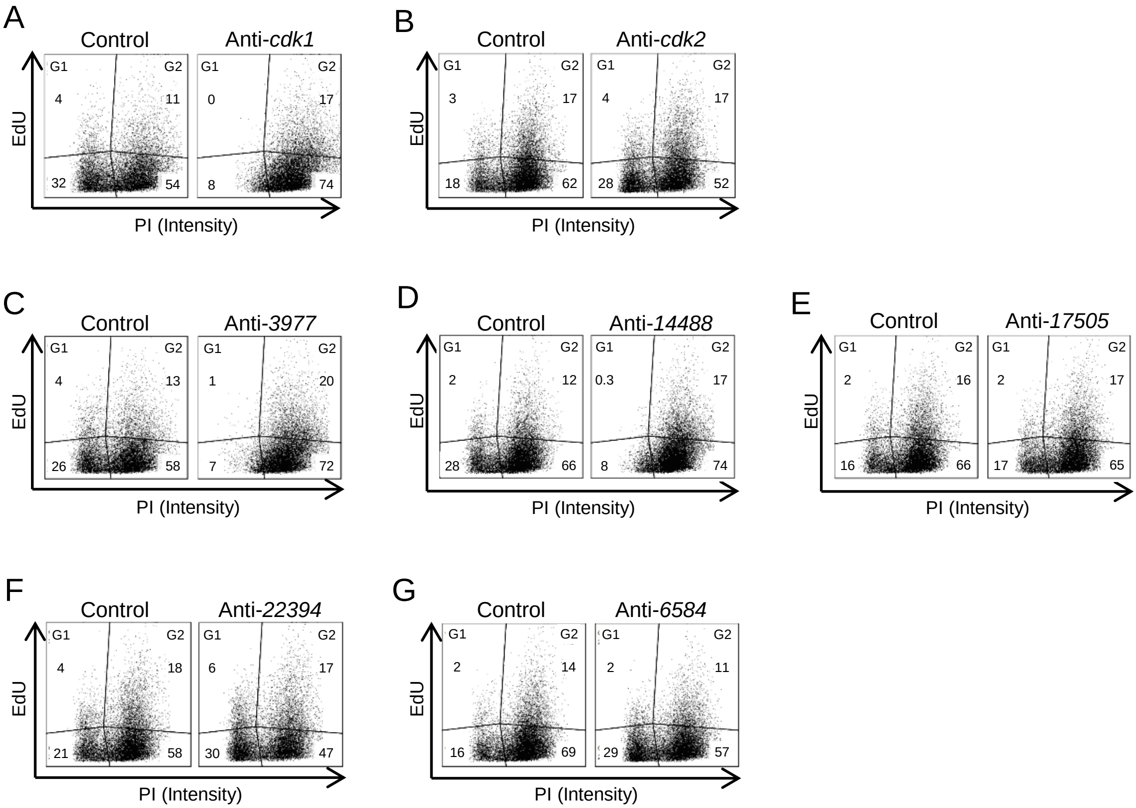

FIG S2 Effects of *G. lamblia* CDK (GlCDK) and *G. lamblia* cyclin (Glcyclin) depletion on DNA synthesis in *G. lamblia*. *Giardia* cells were transfected with (A) anti-*glcdk1*, (B) anti-*glcdk2*, (C) anti-*3977*, (D) anti-*14488*, (E) anti-*17505*, (F) anti-*22394*, or (G) anti-*6584* morpholinos at a final concentration of 100 μM with 50 μM 5-ethynyl-2'-deoxyuridine (EdU) for 6–12 h. The cells were reacted with 1.5 μM iFluor 488 azide and propidium iodide (PI). The DNA content and percentage of cells stained with EdU were determined. Data shown are representative for three independent replicates.

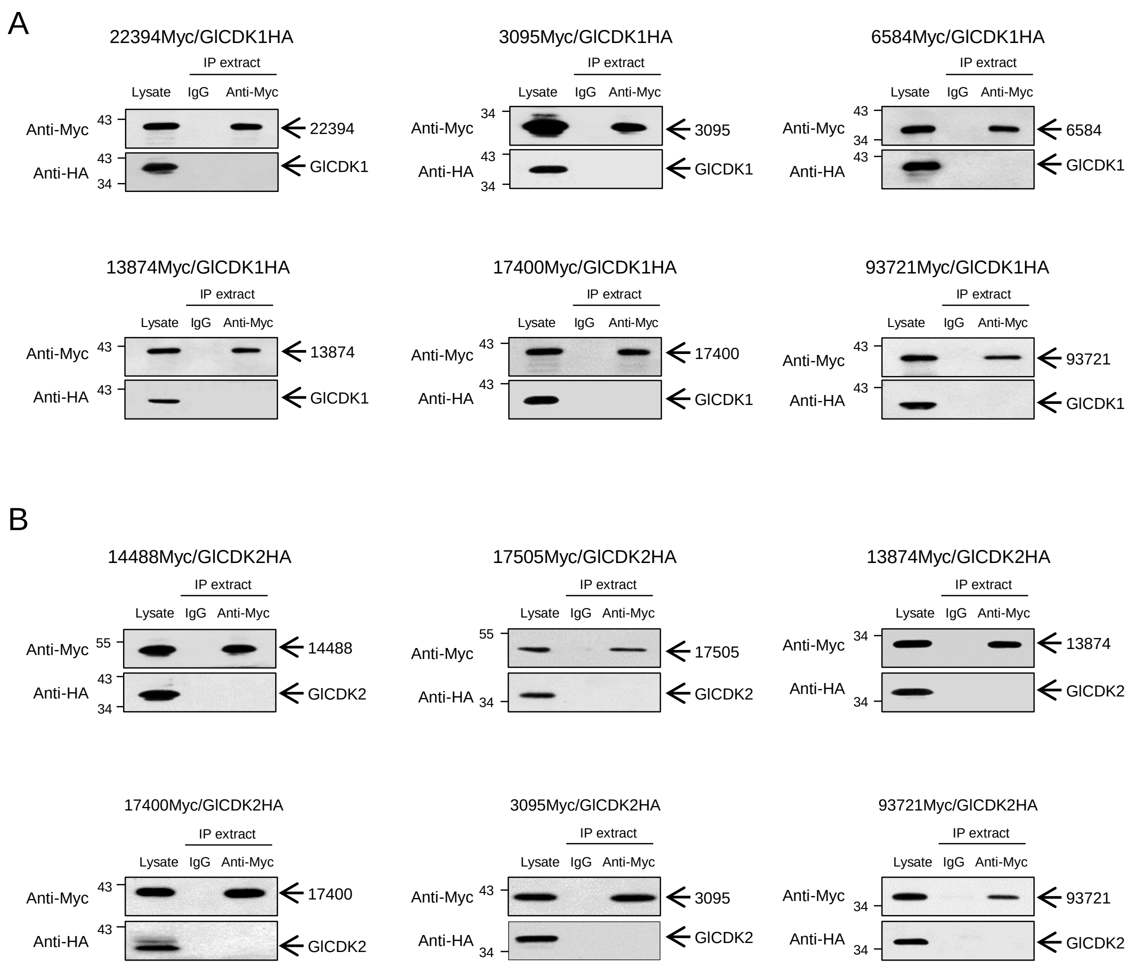

FIG S3 Co-immunoprecipitation of GlCDKs with Glcyclins. (A) Co-immunoprecipitation of GlCDK1 with Glcyclins 22394, 3095, 6584, 13874, 17400, and 93721. *Giardia* cells expressing haemagglutinin (HA)-tagged GlCDK1 and one of the Myc-tagged Glcyclins were co-immunoprecipitated with anti-Myc antibodies, and the resulting precipitates were analysed via western blotting using anti-Myc or anti-HA antibodies. (B) Co-immunoprecipitation of GlCDK2 with Glcyclins 14488, 17505, 13874, 17400, 3095, and 93721. *Giardia* cells expressing HA-tagged GlCDK2 and one of the Myc-tagged Glcyclins were co-immunoprecipitated with anti-Myc antibodies, and the resulting precipitates were analysed via western blotting using anti-Myc or anti-HA antibodies. As controls, the same *Giardia* lysates were included without any treatment or with isotype IgG-agarose.

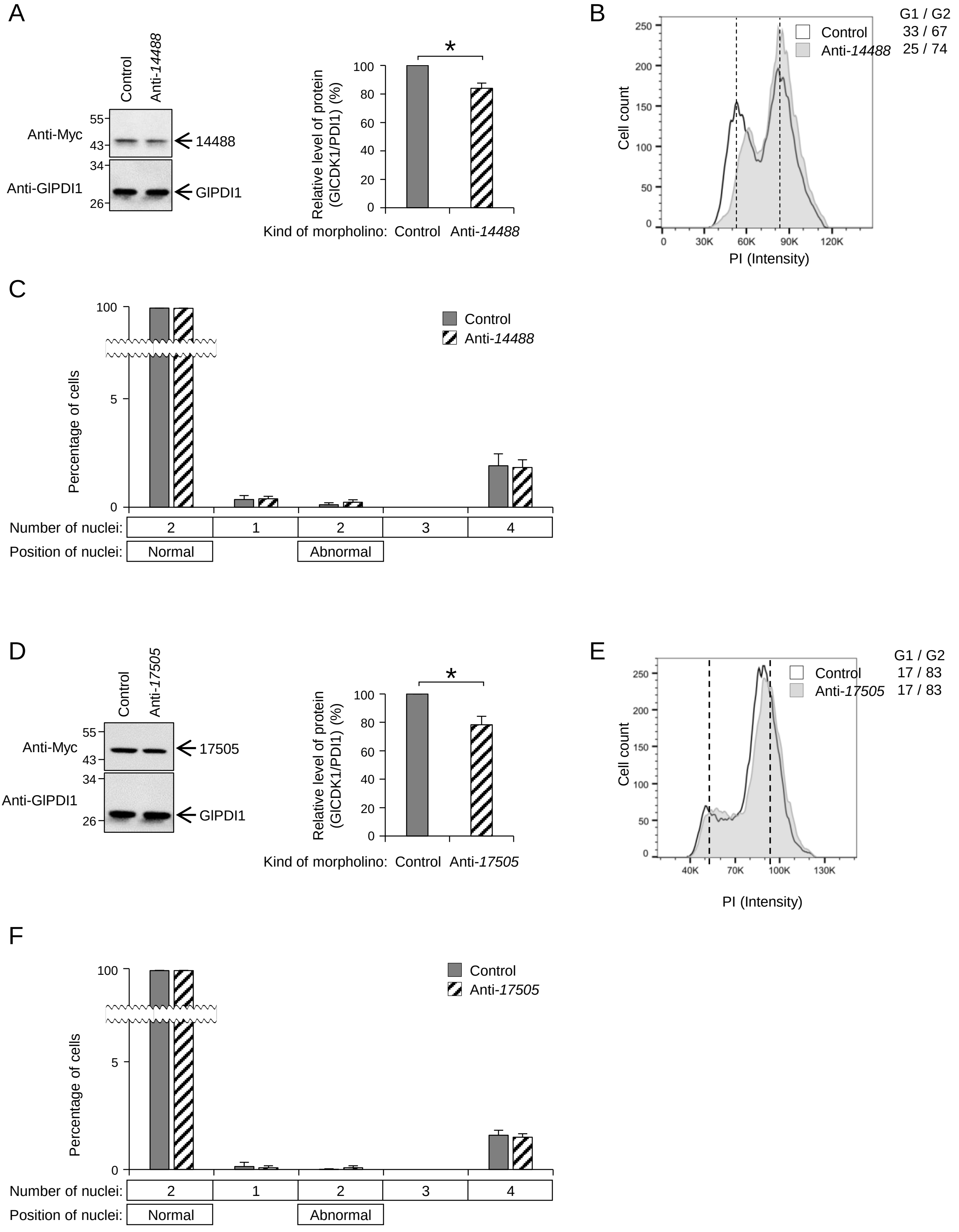
A
Control
Anti-14488
55
43
34
26
Anti-Myc
Anti-GlPDI1
14488
GlPDI1
*
Relative level of protein (GlCDK1/PDI1) (%)
100
80
60
40
20
0
Kind of morpholino: Control Anti-14488
B
G1 / G2
33 / 67
25 / 74
Control
Anti-14488
Cell count
250
200
150
100
50
0
0
30K
60K
90K
120K
PI (Intensity)
C
Control
Anti-14488
Percentage of cells
100
5
0
Number of nuclei: 2 1 2 3 4
Position of nuclei: Normal Abnormal
D
Control
Anti-17505
55
43
34
26
Anti-Myc
Anti-GlPDI1
17505
GlPDI1
*
Relative level of protein (GlCDK1/PDI1) (%)
100
80
60
40
20
0
Kind of morpholino: Control Anti-17505
E
G1 / G2
17 / 83
17 / 83
Control
Anti-17505
Cell count
250
200
150
100
50
0
40K
70K
100K
130K
PI (Intensity)
F
Control
Anti-17505
Percentage of cells
100
5
0
Number of nuclei: 2 1 2 3 4
Position of nuclei: Normal Abnormal

FIG S4 Morpholino-mediated knockdown of GlCDK1-interacting Glcyclins 14488 (A to C) and 17505 (D to F). *Giardia* cells expressing Myc-tagged Glcyclins 14488 or 17505 were prepared at 6 h after transfection with control (grey bars) or anti-*glcyclin* (hatched bars) morpholino. (A and D) Western blots showing Glcyclin knockdown in *Giardia.* Bar graph of relative expression of Myc-tagged Glcyclins in cells treated with anti-*glcyclin* morpholinos compared with that in the control cells. Effect of morpholino-mediated Glcyclin 3977 knockdown on (B and E) DNA content and (C and F) nuclear phenotypes of *G. lamblia*. Data are presented as the mean of three independent experiments. $^{*}P < 0.05$.

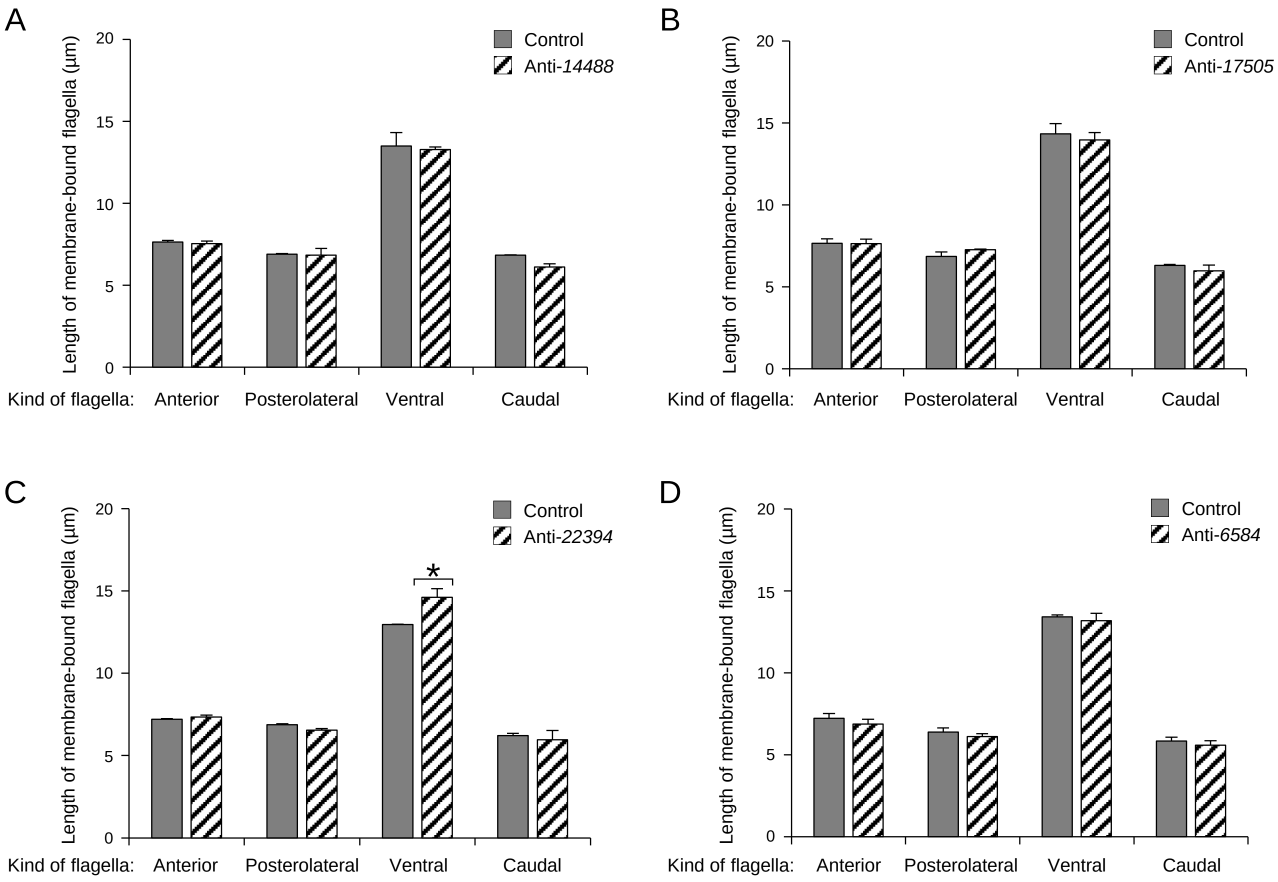

FIG S5 Effects of knockdown of GlCDK1-, and GlCDK2-interacting Glcyclins on flagella length. *Giardia* cells were transfected with (A) anti-*14488*, (B) anti-*17505*, (C) anti-*22394*, or (D) anti-*6584* morpholinos at a final concentration of 100 μM. Flagella length was measured in 35–40 cells per condition. Data are presented as the mean of three independent experiments. *$P < 0.05$.

TABLE S1 Plasmids and strains used in this study

Organism/ Plasmid	Description[a]	Source/ Reference
Giardia lamblia		
ATCC 30957	Clinical isolate	ATCC
Escherichia coli		
DH5α	*supE44, ΔlacU169* (*Φ80 lacZ ΔM15*), *hsdR17, recA1, endA1, gyrA96, thi-1, relA1*	Invitrogen
Plasmids		
pKS-3HA.NEO	Shuttle vector, Amp^R, *neo* gene	(61)
pGlCDK1HA.NEO	pKS-3HA.neo, 927-bp encoding *glcdk1* (GiardiaDB ID GL50803_8037)	This study
pGlCDK2HA.NEO	pKS-3HA.neo, 876-bp encoding *glcdk1* (GiardiaDB ID GL50803_16802)	This study
pKS-3Myc.PAC	Shuttle vector, Amp^R, *pac* gene	(61)
p3977Myc.PAC	pKS-3HA.pac, 341-bp encoding *3977* gene	This study
p14488Myc.PAC	pKS-3HA.pac, 354-bp encoding *14488* gene	This study
p22394Myc.PAC	pKS-3HA.pac, 337-bp encoding *22394* gene	This study
p3095Myc.PAC	pKS-3HA.pac, 166-bp encoding *3095* gene	This study
p6584Myc.PAC	pKS-3HA.pac, 222-bp encoding *6584* gene	This study
p17505Myc.PAC	pKS-3HA.pac, 557-bp encoding *17505* gene	This study
p93721Myc.PAC	pKS-3HA.pac, 250-bp encoding *93721* gene	This study
p13874Myc.PAC	pKS-3HA.pac, 167-bp encoding *13874* gene	This study
p17400Myc.PAC	pKS-3HA.pac, 314-bp encoding *17400* gene	This study

[a] Amp, ampicillin; ^{R}, resistant; HA, hemagglutinin

TABLE S2 Primers used for polymerase chain reaction and morpholino knockdown

Name	Nucleotide sequence (5'-3')[a]
Transgenic *G. lamblia* expressing HA-tagged GlCDKs	
CDK1-F-NotI (GL50803_8037)	CATCGCGGCCGCCGCTCCTCTTGGCTGGAAGCAA
CDK1-R-HindIII (GL50803_8037)	GTTACAAGCTTAGGGTCAAAATCGTTGTGGGAGAA
CDK2-F-NotI (GL50803_16802)	CATCGCGGCCGCTGACGAACGCTGGGTGGTCAGA
CDK2-R-HindIII (GL50803_16802)	GTTACAAGCTTCTTTGCAAAGTACGGATGCTT
Morpholino sequences	
Control	CCTCTTACCTCAGTTACAATTTATA
Anti-*glcdk1*	GCTGCAGACACGCTCATGTTTTTGA
Anti-*glcdk2*	TCATCAAGGTTCGCAGCCGAGCATG
Anti-*3977*	CTGGGATTCGTCGTCTAATGGCATT
Anti-*14488*	GGAGCCCTGCATTCCACAAAGCCAT
Anti-*22394*	TCGCGAATCATGTATTTTTATTCCT
Anti-*6584*	AGGGACGGAGTTTTTTTTCATGTGT
Anti-*17505*	GTCTAGTTTTTGCATAGTGTCGCCA
c-Myc epitope tagged Glcyclins	
3977-F-NotI	GCTAGCGGCCGCGTAAACGGTTTTCCATAACT
3977-R-XbaI	GTACTCTAGACTTTGCTTCCTTTGTATTAAG
14488-F-NotI	GCTAGCGGCCGCAGGCTGTAGCTTAACCTACT
14488-R-ClaI	GTACATCGATTACGGTTGGAGATAATAA
22394-F-NotI	GCTAGCGGCCGCTCCGCATTTTGGAGATCTG
22394-R-ClaI	GTACATCGATATATTTAATACGTTTGGAGG
3095-F-NotI	GCTAGCGGCCGCAATACTTGACTTCATTTGG

3095-R-ClaI	GTACATCGATTTGCAAAGCCTGCGTGACCT
6584-F-NotI	GCTAGCGGCCGCTGATCCTGAGAGCTTCATA
6584-R-ClaI	GTACATCGATGTTATTAAACAACACAACGG
17505-F-NotI	GCTAGCGGCCGCTTGGGACTGAAGCCTAGA
17505-R-ClaI	GTACATCGATACCATCATATCTGTTATTTT
93721-F-NotI	GCTAGCGGCCGCTCTCCGCCGCGTGCAGGCA
93721-R-ClaI	GTACATCGATGCTGCGGACTGGGAGCCTCT
13874-F-NotI	GCTAGCGGCCGCTGATGCCTCGACCCACCTTT
13874-R-ClaI	GTACATCGATATTTGTTTCAACACTCTCTA
17400-F-NotI	GCTAGCGGCCGCTGTCAAGATCGACTAGCATG
17400-R-XbaI	GTACTCTAGACAGCTTCTTTTCCGGCAGAG

[a] Restriction enzyme sites are underlined.
